# Origin of Life on Mars: Suitability and Opportunities

**DOI:** 10.3390/life11060539

**Published:** 2021-06-09

**Authors:** Benton C. Clark, Vera M. Kolb, Andrew Steele, Christopher H. House, Nina L. Lanza, Patrick J. Gasda, Scott J. VanBommel, Horton E. Newsom, Jesús Martínez-Frías

**Affiliations:** 1 Space Science Institute, Boulder, CO 80301, USA; 2 Department of Chemistry, University of Wisconsin—Parkside, Kenosha, WI 53141, USA; kolb@uwp.edu; 3 Earth and Planetary Laboratory, Carnegie Institution for Science, Washington, DC 20015, USA; asteele@carnegiescience.edu; 4 Department of Biochemistry and Molecular Biology, Pennsylvania State University, State College, PA 16807, USA; chrishouse@psu.edu; 5 Los Alamos National Laboratory, Los Alamos, NM 87545, USA; nlanza@lanl.gov (N.L.L.); gasda@lanl.gov (P.J.G.); 6 Department of Earth and Planetary Sciences, Washington University in St. Louis, St. Louis, MO 63130, USA; vanbommel@wunder.wustl.edu; 7 Institute of Meteoritics, Department of Earth and Planetary Sciences, University of New Mexico, Albuquerque, NM 88033, USA; newsom@unm.edu; 8 Institute of Geosciences (CSIC-UCM), 28040 Madrid, Spain; j.m.frias@igeo.ucm-csic.es

**Keywords:** origin of life, Mars, prebiotic chemical evolution, early Earth, astrobiology, CHNOPS, transition elements, sample return, exoplanets

## Abstract

Although the habitability of early Mars is now well established, its suitability for conditions favorable to an independent origin of life (OoL) has been less certain. With continued exploration, evidence has mounted for a widespread diversity of physical and chemical conditions on Mars that mimic those variously hypothesized as settings in which life first arose on Earth. Mars has also provided water, energy sources, CHNOPS elements, critical catalytic transition metal elements, as well as B, Mg, Ca, Na and K, all of which are elements associated with life as we know it. With its highly favorable sulfur abundance and land/ocean ratio, early wet Mars remains a prime candidate for its own OoL, in many respects superior to Earth. The relatively well-preserved ancient surface of planet Mars helps inform the range of possible analogous conditions during the now-obliterated history of early Earth. Continued exploration of Mars also contributes to the understanding of the opportunities for settings enabling an OoL on exoplanets. Favoring geochemical sediment samples for eventual return to Earth will enhance assessments of the likelihood of a Martian OoL.

## 1. Introduction

The history of the proposition whether there is life on Mars has been a roller-coaster of disjoint conclusions. Early conjectures by astronomers working at the limits of observability included technosignatures (canals) and seasonal vegetation patterns (“wave of darkening”). More modern studies cast doubt, and the initial space exploration of Mars with snapshot images during fast flybys left the impression that the red planet was far more Moon-like than Earth-like (Mariner 4, 6, 7). Although the residual water ice cap was observed in the south polar region and water vapor detected in the sparse atmosphere [1], the crater-littered surfaces and lack of evidence of a substantial hydrosphere cast further doubt as to whether Mars was ever habitable.

Despite its short useful life after the subsidence of a global dust storm, the Mariner 9 orbiter imaged numerous geological features, such as braided and networked channels, which attested to stages of fluid erosion, including flowing water and runoff [2], notwithstanding the confirmation that the contemporary climate of Mars is too cold and too dry for the significant persistence of liquid water. The Viking missions verified the cold, dry surface and discovered a global soil that was lacking in organics or any firm indications of metabolic activities [3]. Worse, the global soil was virtually identical in geochemistry and chemical reactivity on opposite sides of the planet. Mars was bland, and uninteresting. This idea prevailed for 20 years, during which no missions were sent to Mars.

Following this long hiatus, subsequent missions to Mars ranging from highly instrumented orbiters to long-lived rovers have radically changed that view of uniformity. Orbiters have detected mineralogical diversities across the planet, while the Mars Exploration Rover (MER) and Mars Science Laboratory (MSL) rovers have revealed even greater diversity at the local scale. These missions have broadened and deepened the understanding of the changes in climate and history of liquid water [4], while also vastly increasing the knowledge of the chemical diversity [5,6] and the sedimentary history [7] of Mars.

Although much has been discovered concerning the habitability of Mars (e.g., [4,5,6,7,8]), an equally important consideration is whether the environments on Mars were just as conducive to the abiotic origin of life (OoL) itself. Could life have arisen on Mars? [9,10,11,12,13,14,15,16]. A hallmark of a biosphere of Life is its ability to adapt its forms such that it has species and strains of organisms which variously are able to survive and even sometimes prosper in nearly all conceivable aqueous environments. Additionally, many hostile environments which are intermittent can be tolerated until more suitable conditions arise. In contrast, prebiotic chemical evolution (PCE), leading to the first life form and creation of a biosphere, is more likely restricted to not only certain limited conditions but may even require a specific sequence of special environmental changes for it to succeed [13].

In addition to liquid H_2_O, for an OoL, the starting organics, essential elements, and access to energy sources are needed. We shall, therefore, examine the extent to which Mars as a planet may have been able to supply these specific conditions during its existence, including the past, the present, and the future. Although the OoL on Earth is itself not completely understood, we may use the current state of knowledge to constrain the possible precursor environments, chemistries, and materials that would have been necessary on Mars for a similar origin.

From homogeneous magmas, brines, and atmosphere come settings which host a wide diversity of mineral grains, evaporite salts in sequences, aqueously altered minerals, as well as raindrops, snowflakes, and micro-climates. Homogeneity can lead to heterogeneity, especially when driven by changes in temperature, pressure, chemical environment, and other factors. Life similarly arises, but a variety of hypotheses exist which envision different physical and chemical conditions under which it can arise or has arisen. The extent to which these variously conceived conditions were available on Mars is a topic worthy of attention because it affects the likelihood that life could arise ab initio, perhaps even more than once, in any given planetary system. By examining conditions on Mars over deep time, a better picture of the early environments on ancient Earth may become apparent and, therefore, the search for life or prebiotic evolution is inexorably linked to the search for our own origins on Earth. The rover missions, and the MSL Curiosity rover in particular, have made many discoveries which greatly increase the prospect that life could have arisen on Mars.

## 2. Materials and Methods

Some concepts for the origin of life are focused on the prebiotic organic chemistry which must take place to create the complex functions and structures that are associated with living entities. Other hypotheses focus on the environmental conditions that nurture such activities, including the sources of the ingredients and energy that are needed, and certain specific settings in which the OoL could occur.

We shall summarize the breadth of these concepts and their chosen settings, noting the common and unique aspects of each. In the subsequent sections, the current knowledge of the status and history of Mars will be assessed for its compatibility with these various scenarios.

The most fundamental recognized requirement for life is the medium of liquid H_2_O. Liquid water provides multiple favorable physicochemical factors, including its solvation capabilities, wide temperature range, polar properties, and so forth, as well as enabling the mobility of its constituents while, overall, being contained. Furthermore, it is biochemically essential, with one-third to one-half of all metabolic reactions having H_2_O molecules as either reactants or products [17]. All these properties of water are also at play in PCE scenarios. It is, therefore, the most fundamental component needed for life as we know it (LAWKI).

### 2.1. Organic Molecules and Elements

The standard compositional requirement for life is for the elements carbon, nitrogen, etc. making up the CHNOPS group. These include the primary formation elements (CHONS) which make up the amino acids, and hence proteins. In certain other critical biochemicals, sulfur does not play a principle role but phosphorus does: RNA, DNA, phospholipids, ATP, and other constituents. Unless a potentially habitable environment has these six elements available, LAWKI is not possible.

**Figure 1:**
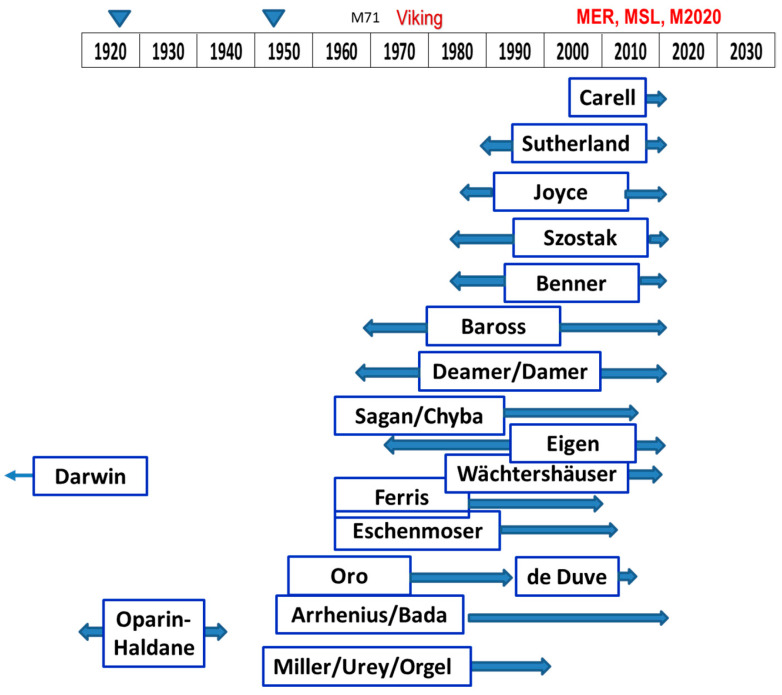
Some example groups that have spearheaded investigations into the origin of life, showing accelerated intensity in the study of prebiotic organic evolution pathways, subsequent to the findings of Miller–Urey experiment in 1953 [18] (Each block is one decade, e.g., 2010 = 2010 to 2019).

Laboratory studies of prebiotic chemical evolution have accelerated in recent decades, with many promising results. To reflect this progress, some of the groups spearheading various investigations are portrayed in the rough timeline of Figure 1. Their findings have identified several key pathways from simple organic compounds to the classes of complex molecules utilized by extant organisms. They also have evaluated many candidate reaction sequences which either do or do not produce significant yields, or have very specific requirements that may or may not be plausible in the early times of a lifeless planet.

Following the discovery that amino acids could be formed by lightning-like electrical discharges in a mixture of simple gases [18], numerous laboratory investigations have sought to elucidate natural processes and reactions leading to PCE that could occur on early Earth.

Early work by de Duve [19] focused on the high-energy bond in thioesters formed by combining simple organic molecules, volcanic H_2_S, and carboxylic acid molecules. His observation was that thioesters are deeply ingrained in many pathways of contemporary metabolism and could have been a precursor to the use of the high energy phosphate bonds. In biological systems on Earth, the energy-rich thioester bond provides the energy source for the phosphorylation of ADP to ATP, which makes sulfur-based energy management indispensable [20]. However, there are many metabolic pathways that operate completely independent of ATP, relying on acetyl-CoA to drive reactions, including the biosynthesis of fatty acids. The structure of acetyl CoA includes the relatively simple sulfur-containing molecule cystamine at the end where the thioester bond would be, as well as, at the other end, the relatively complex 3′-phosphorylated ADP, which acts as a handle for molecular recognition and attachment. This structure supports the notion that thioester sulfur chemistry might have progressed, independent of a phosphate-based system.

A one-pot synthesis of thioesters using FeS and peroxysulfate or UV stimulation has been demonstrated without the need for enzymes [21]. A combinatorial analysis of coupling of redox reactions to thioester PCE has led to an “organo-sulfur” protometabolism hypothesis [22]. Thioesters may have played a role in peptide ligation, as organic catalysts rather than requiring metal cofactors [23].

The “iron-sulfur world” analyzed by Wächtershäuser [24,25,26] proposes catalytic properties of Fe-S for forming organic compounds, and takes advantage of higher temperatures and sulfide availability at hydrothermal vents. Often using CO, thiols, and metal sulfide catalysts, these reactions can form activated acetic acid with steps similar to those of the Wood–Ljungdahl carbon fixation and acetogenesis pathways [27]. Thioesters formed abiotically can be partially protected from subsequent hydrolysis through incorporation into vesicle walls [28]. Abiotically formed thioesters might have been the foundation of the Earth’s origin of life (e.g., [24,29]) and/or the basis of early cellular metabolism using a limited set of genes [30].

The Sutherland group has shown that a milieu of HCN (and some simple derivatives), plus sulfur species and a Cu catalyst can variously produce the precursor molecules for all three of the most fundamental classes of biomolecules: RNA, proteins, and lipids, with just simple variations in environmental conditions [12,31,32,33,34,35]. This set of reactions, referred to as a “cyanosulfidic” protometabolism, also requires UV light, as well as Fe, Ca, and P. This group of investigators has envisioned a set of bodies of water with interconnecting streams to produce serial syntheses of ever more complex assemblages of the precursor molecules needed for a protocell.

Cyanide destruction by reactions with H_2_O and/or HCHO can be prevented through formation of ferrocyanide and salts thereof, with subsequent thermal decomposition to restore the availability of cyanide feedstock for PCE [12,36].

It has now also been shown that the S-containing amino acid, cysteine, can catalyze the elongation of peptides [37]. Incorporation of cysteine monomers at strategic locations in the amino acid chain of a protein allows the formation of disulfide covalent bonds which rigidize the protein in its optimum three-dimensional configuration for its powerful enzymatic activity.

The Carell group has demonstrated [38,39,40,41] the synthesis of all four nucleotides for RNA from just ribose (formed elsewhere) and other small molecules (HCN, NH_3_, nitrate), using FeS, sulfite, Cu, Zn (or Co), B, and carbonate, with the help of wet–dry cycling and selective changes in physical conditions (temperature, pH). This can be done with the same simple ingredients (“one-pot”) and is another breakthrough in finding likely PCE pathways [40].

Suggestions for boron participation in prebiotic chemistry has a significant history [42]. Benner and colleagues have investigated the use of boron minerals in the stabilization against rapid degradation of the key sugar, ribose [43,44,45], as well as pointing out other ways in which mineral versions of this element are useful for prebiotic evolution [42,44,45,46]. With Ni^2+^, borate, urea, and a cyclic phosphate, they demonstrate the formation of ribonucleotides [47]. It is even proposed that perhaps Mars is a more likely locale for OoL than early Earth because of its arid environment, which is favorable to wet–dry episodes and concentrations of borate and other minerals [10]. They also invoke the protective effect of magmatically released SO_2_ in producing a stable sulfonate derivative as a temporary storehouse of labile HCHO during PCE [48].

Earlier, the Ferris group had shown that oligomers of RNA could form under the catalytic action of montmorillonite clays on ribonucleotides [49,50].

The acetyl CoA (Wood–Ljungdahl) pathway is the most primitive and simplest sequence to formation of important organic molecules via CO_2_ fixation with H_2_, and can be catalyzed by native Fe°, Ni°, Co° and also to some extent by Mo° or W° [51]. This overall exergonic reaction can also be catalyzed by Ni_3_Fe (awaruite) [52]. In microbes, its enzymatic infrastructure includes cofactors of Co, Ni, Fe and S. This pathway is only one of at least six different pathways in the world of biology by which CO_2_ fixation proceeds. It is the simplest and also the only one which can also be accomplished without enzymes, plus the only one that can have a net generation of ATP rather than a consumption of ATP [52]. Hence, it is often considered as one of the most rudimentary, fundamental, and earliest forms of metabolism [27,52].

Deamer and collaborators have championed the amphiphilic compounds extractable from carbonaceous meteorites for their ability to form vesicles, and the role these might play in the very earliest PCE by enabling the compartmentalization of key components that go into making up the system that the protocell needs for its primitive functions [53].

High concentrations of salts, including Mg^2+^, are found to destabilize the formation of vesicles from amphiphilic precursor molecules [53]. It has, therefore, been proposed that subaerial hot springs may be more conducive to the OoL than deep oceanic hydrothermal vents (OHV) because typical spring waters have lower salt concentrations, especially for Mg^2+^, than the seawater environment surrounding OHVs (with example spring waters concentrations of 0.03–3 mM Mg^2+^ compared to 53 mM for seawater and 9–19 mM in the Lost City white smoker [54]). However, it is now also found that certain biological amino acids can stabilize fatty acid membranes, even in the presence of high ionic strength, including solutions such as NaCl and Mg^2+^ salts [55]. Another substitute for Mg^2+^ is Fe^2+^ ions in solution [56], if they can be prevented from becoming oxidized. An organic chelator, citrate, still allows for RNA replication enhanced by Mg^2+^, but prevents the Mg disruption of vesicle formation while enhancing their permeability to RNA tetramers [57]. Coacervates and other encapsulation pathways are also being investigated [58].

The Deamer and Damer group describes hot springs in a volcanic environment as the setting for the origin of life [15,54,59,60,61,62]. This group has also emphasized the wide-ranging benefits of wet–dry cycles to promote dehydration polymerizations of a wide variety of monomers [60], including RNA, DNA, and proteins.

Progress continues to be made, for example by the Joyce group [63,64], in test-tube evolution experiments of ribozymes in high levels of Mg^2+^ to evaluate the RNA World hypothesis for the origin of the genetic basis for life [62,63,65,66,67].

Transition Elements. For the management of the extraordinarily complex chemical pathways in a metabolic system, the organism must orchestrate reactions by means of catalysts. In the organisms which have emerged as the biosphere has proliferated and evolved over geologic time, exquisite protein-based enzymes have emerged, about 40% of which employ one or more of the transition metal elements or divalent cations from group 2 in the periodic table (Mg, Ca) [68]. The group 4 transition elements, Ti and Zr, are relatively abundant minor and trace elements, respectively, in basaltic materials, but have had little if any significant biological involvement. However, the first row transition elements in the Period Table subsequent to Ti, namely V, Cr, Mn, Fe, Co, Ni, Cu, and Zn, are known for their various catalytic properties and as essential elements in many if not most biological systems [68,69]. They have also been proposed as essential progenitors to an origin of life [70]. Additional key catalytic elements include Mo and W, which are lower-row transition elements in Group 6 along with Cr, which is not involved with living systems to any major degree. However, Cr^3+^ (in addition to Zn^2+^ and Fe°) has been shown to promote certain reactions of the reverse Krebs cycle, whose origins are also suggested to have been an early anabolic biochemical pathway for CO_2_ fixation using H_2_O [71].

Metalloenzymes presumably were gradually evolved to their current extraordinarily efficient form [72], but it is hypothesized that for some, or many, the metal ions themselves may have been the original primitive catalysts [69,71,73,74].

Although these trace elements are needed, it is at levels that are species-specific. Each element has a minimum concentration and a maximum acceptable level before toxicity or stress sets in. Any given species or strain is adapted to certain environments, and generally has sensing, as well as active transport systems which import or export elements as needed for optimum metabolic functionality [68,69,75].

Early peptides would have had important interaction with metal ions for various functions, including Mg, Zn, Fe-S, Cu and Mn [76]. It has been hypothesized that primitive oligopeptides were functional with only four amino acids (Gly, Ala, Val, Asp) which have specific domains, the binding metals of which range across Mg, Mn, Zn, and Ni [77].

One of the most important enzyme cofactors is a combination of ions, the [FeS] clusters which are fundamental to electron storage and transfer. Proteins such as the ferredoxins, which participate in photosynthesis, nitrogen fixation, and assimilation of hydrogen, nitrogen, and sulfur, rely on such clusters. There are [FeS(Ni)] clusters in hydrogenases, which promote oxidations of H_2_, one of the most important catalytic functions in the biosphere.

Molybdenum plays several important roles in extant organisms, including the extraordinarily important nitrogenase enzyme ([MoFeS] cluster) in diazotrophic organisms for the fixation of atmospheric dinitrogen. Although Mo is the common cofactor, there are also vanadium-based nitrogenases, and even an Fe-only version. Several members of the Euryarchaeota phylum of the Archaea can fix nitrogen without Mo (possibly indicating a more rudimentary function), whereas members of the Crenarchaeota phyla utilize Mo and Cu for nitrification and denitrification, respectively [74].

Mo-containing enzymes are also involved in nitrate and sulfate metabolism. Because of a lack of the sufficient availability of soluble Mo prior to the Great Oxidation Event [78], other elements, such as tungsten and vanadium, may have provided the needed catalytic activity for these metabolic functions.

The composition of an ancient metallome from 3.33 Ga carbonaceous residue in the Barberton indicates the participation of Fe, V, Ni, As, and Co in that example of early organisms. This residue was also modestly enriched in Mn, Cu, and Zr, but with an absence of detectable Mo and Zn [79].

Mulkidjanian [80] has emphasized the role of zinc ions, among others, in the progress toward biological activity. Although zinc today is not essential in all procaryotes, it is prominent in many functions in eukaryotes and the hormones of their multicellular forms, with special use of the “zinc finger” proteins. Altogether, Zn in proteins may perform as many as six different general functions, but was presumably was only sparsely available on early Earth because of its insolubility as ZnS [69]. On a planet where Zn is readily available, one or more of its potential functions may be broadly utilized in early metabolism, such as the one-pot synthesis of nucleotides [38].

Another role of transition elements, especially for Zn and Ni, is their enhancement of adsorption of nucleotides onto montmorillonite and nontronite clays [73].

### 2.2. Energy Sources

Overcoming the entropic barriers to life’s high degree of organization requires inputs of energy, as do many of the chemical reactions of its metabolism. In addition to the usual energy sources, such as heat, sunlight (including UV), ionizing radiation from galactic cosmic rays (GCR), and lightning, there are the chemical disequilibria that result when high-temperature magma releases volatiles or is quenched, as well as when air and water react with regolith, rocks, and each other.

Chemotrophs can live solely from the energy of chemical reactions. The variety and, in many cases, versatility of chemotrophs to obtain energy from many different redox couples that result from geochemical and atmospheric processes continues to amaze. For example, an evaluation of the Gibbs energy for 730 redox reactions among 23 inorganic reactants (from 8 elements: CHNOS plus Fe, Mn, As) possible in a shallow-sea hydrothermal vent found that almost one-half were exergonic reactions [81]. Intricate syntrophic relationships between communities also allows for the extensive exploitation of multiple sources of latent chemical energy in the environment. The extraordinarily high level of sophistication in metabolic processing of various redox couples by microbial species, especially in the archaea, would not be fully available to processes of prebiotic chemical evolution, so it is necessary to identify specific appropriate energy sources for the OoL.

### 2.3. Planetary Settings

Beginning with Darwin’s musing that life may have started with some “warm, little pond” with ingredients of N, P, and energy, there have been pond scenarios for an OoL. These days, the feedstocks usually contemplated are molecules residing in a reducing atmosphere with CH_4_, NH_3_, and/or H_2_. From the energetics of lightning and its chemical products, a variety of organics, including amino acids [18], are produced. Others invoke rare but fortuitous contributions of pre-formed organics directly into ponds from sources, such as comets [82] or carbonaceous meteorites [83]. From the interplay of several factors, ponds of intermediate size are the more likely settings than larger or smaller bodies of water [13].

Because of the multitude of biochemicals that make up a living system, it is envisioned that ponds and streams must undergo changes that bring together different ingredients. Although most PCE experiments are conducted at circumneutral pH, there are some which benefit or begin from a more acidic, or more basic, environment [14,33]. RNA stability increases with acidic pH and with Mg^2+^ rather than Fe^2+^ in solution [16].

As a setting or combination of settings with spatial and temporal variations progresses from prebiotic chemical evolution to replicating molecules, vesicles, and orchestration of catalyzed metabolic functions, it enables the protocell. Further development can lead to the colonizer cell to complete the transition from being a wholly inanimate locale to one which contains the earliest forms of life which can potentially establish a biosphere. This is termed the macrobiont [13] because this setting is macroscopic in scale yet contains molecular- and microbe-scale entities which have biological capabilities. Before this organizational transition to the birth of life, such a potentially appropriate setting for an origin of life is simply a proto-macrobiont.

A quite special type of setting is the oceanic hydrothermal vent (OHV), usually in a group comprising a vent field. These “smoker” chimneys exude a rich variety of constituents extracted by seawater circulating through hot basalt beneath the seafloor at magmatically active locations, especially at tectonic-plate boundaries. A variety of possibilities for an origin of life have been addressed for the vent chimneys which form from effusing streams [84,85], including porous cavities that trap components [86] for reactions [87] and can concentrate products by thermogravitational trapping [88]. Recent attention has moved from the acidic black-smokers to the much less abundant alkaline white-smoker chimneys because of the production of H_2_ by the latter via the serpentinization reaction between H_2_O and ultramafic minerals [84,85,87,89,90]. With the high pressures that the deep sea affords, H_2_O can remain liquid at temperatures of several hundred °C, which shortens the time, increases the efficiency, and enhances the extractions of soluble ions into the stream. These effusions are rich in sulfides, especially those containing Fe, Cu, and Zn. Sulfide-rich bubbles with Fe and Ni, produced by seepage from vent fields, have been proposed as a micro-setting for PCE leading to life [29,91].

Smokers also include other bio-essential elements, such as Mo, Co, Ni, Se, as well as additional elements [92] not generally associated with the needs of living organisms or the OoL. The vent fluids’ dissolved loads enable a diverse, but highly localized macrofauna by providing not only these essential nutrient elements, but also chemical energy from abundant and diverse redox couples.

In addition to sub-oceanic settings, there is strong interest in subaerial settings where hydrothermal activity is prominent [15,54,61,93]. These include hot springs, geysers, mud pots and other manifestations in geothermal areas, driven by shallow magmatic chambers and groundwater.

Although transient, the energy deposited during the impact cratering process into wet or permafrost-laden regolith to form warm crater lakes with subsurface hydrothermal activity has been a scenario of increasing interest as a macrobiont for the OoL [94,95,96]. The Chicxulub impact crater on Earth includes abundant evidence of creating a hydrothermal system and supporting colonies of sulfate-reducing organisms for ~3 million years [95]. Additionally, craters as small as ~25 km diameter may produce buried hydrothermal activity which persists for ~1 Myr, while ~5 km craters could result in hydrothermal systems that persist for thousands of years [96].

Another model ties the origin of life to a single cataclysmic event [97], the impact of a planetary-scale object which is large enough to have a native Fe core, which then reduces available H_2_O to create a H_2_-rich atmosphere. While causing a transient environment that would be sterilizing and also delivering the siderophile elements that are in the Earth’s mantle, its aftermath is proposed to enable the origin of the RNA world.

Wet–dry and freeze–thaw cycles. Several decades ago, there began the search for physical processes which could promote certain chemical reactions which do not proceed well in water. These reactions include dehydrations to enable polymerization. One class is the formation of oligopeptides from amino acids and another is the synthesis of polynucleotides (RNA, DNA) from their constituent nucleotide monomers (ATGUC). Although modern cells routinely perform these functions with the help of enzymes and ATP energy, the challenge for PCE remains. There are a few paths to these results using “condensing agents”, but these are generally not plausible natural chemicals. What was learned in these early studies was that thermal cycling can help promote polymerization reactions, but drying a prebiotic milieu and then re-wetting, followed by more cycles, is an even more effective way to promote the dehydration to form oligomers [40,46,60,98,99,100,101]. Similarly, freeze–thaw cycles can promote ligations for an RNA world [102,103,104,105]. Much additional study has demonstrated the general and powerful applicability of these plausible natural oscillations in environmental conditions to achieve highly relevant results for PCE [93].

River deltas are of considerable interest because they intrinsically include braided streams which enable ponds that are intermittently semi- or totally isolated, and then brought into communication and mixing as outflows change in volume or rate. The Gilbert-type delta in Garu crater on Mars is estimated to have developed over a period of as much as 10^5^ years [106]. However, deltas can also form and desist rapidly, as in the <1 kyr estimate for the fluvial delta in Jezero crater [107], which may be too rapid for relevant chemistries to successfully proceed.

Subaerial semi-arid environments, including geothermal areas, are ideal for generating repetitious wetting and drying. Ponds in cool environments, especially along their foreshores, can experience both wet–dry and freeze–thaw events. Mudflat patterns can create further heterogeneity by trapping pond constituents temporarily in juxtaposed mini-environments [13] which could allow semi-independent progressions along the PCE arrow toward life with subsequent flooding to bring them together.

Porous sediments enable the phenomena of geochromatography to separate constituents. In this scenario, PCE components move along aqueous gradients containing heterogeneous concentrations of anions and cations, allowing for the separation of organic molecules based on size and charge [108]. Furthermore, in such a scenario, the concentration of anion and cations could also provide environments of differing water activity, effectively becoming a pseudo “wet and dry” environment. A feature is that such an environment could provide natural compartmentalization of molecules based on hydrophilic/hydrophobic reactions.

## 3. Results

Although Mars has many specific differences from Earth (10× smaller mass, 200× less atmosphere, 6× to 20× less H_2_O per unit surface area, 50× less volcanism, and no plate tectonics or spreading centers), it nonetheless has many, perhaps all, of the ingredients needed for various scenarios proposed for the origin of life. The relevant comparison must be made with Earth itself, not the known or envisioned exoplanets, because Earth is demonstrably the only locale where life did actually once successfully originate (unless it came via lithopanspermia from Mars itself [10]).

The key properties of an incipient macrobiont include the availability of essential ingredients in terms of water, key elements and molecules, access to energy sources, and existence of the variety of local settings with physical and chemical properties which have been suggested to be adequate or essential for the processes leading to life. Sources that are intrinsically available on Mars as well as exogenous sources of ingredients (comets, asteroids) could each contribute.

### 3.1. Elements and Molecules on Mars

At its surface, current-day Mars offers an oxidizing environment [109,110,111,112]. This is contrary to what generally would be desired for most reactions in PCE [9]. However, both early Earth and early Mars are now thought to have had abundant atmospheric species in reduced form, such as H_2_ and CH_4_, in order to achieve a sufficient greenhouse effect to enable liquid H_2_O at their surfaces [4,113,114,115].

The primary igneous minerals of Mars generally mimic those prominent on Earth, especially those in basalts, including olivines, pyroxenes, feldspars, apatites, etc. It is the mafic minerals, olivine and pyroxene, which can react with H_2_O at moderate to high temperatures to produce H_2_ in the reaction to form serpentines and magnetite [116] and, by additional reaction with CO_2_ to produce methane [110]. These serpentinization reactions can also liberate important minor and trace elements from the crystal latices of these minerals.

#### 3.1.1. Organics and CHNOPS Elements

Not only are these CHNOPS and other elements essential to all life as we know it, but their molecular or mineral form is important because it affects reactivity and availability in aqueous media (e.g., valence state; solubility).

Carbon. From magmatic outgassing, the early Martian environment could have hosted significantly greater quantities of CO_2_ in its atmosphere. The total inventory of CO_2_ is estimated to have been in the range of 1 to 3 bar [117], based on loss rates observed by the MAVEN mission and orbital observations of carbonates on Mars, or less than 1–2 bar [118,119] when based on the size–frequency distribution of the ancient craters. The current nominal partial pressure of CO_2_ on Mars is 6 mbar (plus traces of CO). Compared to Earth’s 0.4 mbar of CO_2_, this is more than adequate to support the biosynthesis of the array of organic molecules needed by living cells across a broad biosphere.

Both planets are thought to have had atmospheres with CO_2_ more in the 1+ bar range, in order to have a sufficient greenhouse effect to prevent runaway freezing [115,120]. The Earth’s higher rate of magmatic devolatilization has resulted in massive carbonate deposits, equivalent to tens of kilobars of CO_2_ when integrated over geologic time [121].

Other sources of carbon on an emerging world can come from exogeneous inputs, such as comets, carbonaceous meteorites, and interplanetary dust particles. Compared to carbonate and CO_2_, an advantage of these sources is that the carbon will be in relatively reduced molecular forms important to PCE, such as hydrocarbons, N-rich organics (including cyanide), and even abiotically synthesized amino and carboxylic acids [122].

Although Mars has a 72% smaller cross-section for impact than Earth and a weaker gravity field, its cross-section for impacts scales the same as its surface area, such that the density of craters on land could be very similar. At the location of Mars farther out in the Solar System, it would be expected to experience a greater flux of C-bearing impactors by being nearer to the organically enriched bodies in the outer asteroid belt, as well as Kuiper belt objects and comets. Furthermore, from the relatively high Ni content of the Martian global soil (ranging from 400 to 600 ppm [123,124]) compared to the Ni in Martian basalts (165 ppm in Adirondack, 81 ppm in BounceRock, 79 ppm in Shergotty meteorite [125]), it can be inferred that the contribution of these exogeneous delivery sources to the shallower regolith of Mars resulted in relatively higher concentrations of organics than was for Earth, which had more extensive regolith turnover and obscuration due to higher rates of extrusive volcanism and sedimentary processing. The expanse of Earth’s global-scale ocean would have also lessened the relative importance of these endogenous contributions because its high dilution factor likely reduced their concentrations to be low, or negligible.

Organic compounds, such as HCN, may be produced in a reducing atmosphere by various processes [34]. These include production by photochemical or ionizing radiations reactions [126], by lightning [115], and even some by ionization during atmospheric passage of hypervelocity bolides and their impact ejecta [127]. Cometary delivery has long been considered a potential major source of HCN [128,129].

Hydrogen. This element mostly occurs in its oxidized form, as H_2_O. Although water is the foremost use for H atoms to support both life and the origin of life, some must also be available to form organic molecules. For example, the dry mass of a typical microbe includes 1.77 H atoms per C atom [130]. Likely forms of H in the early atmospheres include not only H_2_O vapor, but also reduced molecules such as H_2_, CH_4_, NH_3_, HCN, and H_2_CN_2_. As noted above, water molecules can be decomposed to give off H_2_ through the serpentinization reaction, but there are also other sources of H_2_ early in Mars history [116].

Little direct evidence is available for the presence of significant H_2_ on either early Mars or Earth. However, the extensive hydrologic activity now abundantly evident for the earliest history of Mars mandates the presence of a stronger atmospheric greenhouse effect than currently, and the best solution of that uncertainty is if Mars had an early ~1 or 2 bar CO_2_ atmosphere but combined with 1% to 20% of H_2_ gas [113,131], with similar scenarios for the early Earth [115]. Modern models which implicate reducing atmospheres for these planets negate the arguments at one time raised against the theories of Haldane and Oparin, as well as the pioneering experiments by Miller and Urey [18], all of which presumed a more reducing rather than oxidizing atmosphere early in our planet’s history when the OoL was occurring.

Nitrogen. This element is primordially expected to be gaseous at the surface, in the form of the relatively inert N_2_. The current Martian atmospheric level is only 0.16 mbar, about 5000× lower than on Earth. However, the majority of terrestrial organisms cannot metabolize dinitrogen gas, and instead must obtain this element from ammonium salts, nitrates, or nitrogenous organic molecules in the soil. A few microorganisms, the diazotrophs, are able to “fix” N_2_ gas into one or more of these compounds. These organisms utilize Fe-S-Mo- or V-based nitrogenase enzymes to accomplish these conversions.

A Martian biosphere may have been limited in its vigor by this shortfall of N, just as many ecosystems on Earth are often growth-limited by the availability of nutrient N. However, nitrates have now been found in Martian soils [132,133]. On early Earth, prior to biological N_2_ fixation, nitrates and nitrites formed by lightening may have been the source of N needed for prebiotic chemical evolution [134]. The nitrate ion was sought in the Wet Chemistry Laboratory (WCL) soil water experiment on the Phoenix mission [135,136] but was not found at a detection limit that would be equivalent to 25 mM (if at a 1:1 W/R ratio). However, nitrate has been detected by the Evolved Gas Analyzer (EGA) in certain Gale crater soils, albeit at only ~5 µM equivalent (300 ppm), and up to about 3× this amount in some samples [132]. Organic nitrogen compounds (pyrroles and imines) were found in the Tissint Martian meteorite, linked to electrochemical reduction on mineral surfaces [137]. The reduction of N_2_ to NH_3_ is a well described process in electrochemistry using Fe°/magnetite [138] or pyrite [139] as electrocatalysts.

Due to its rarity, the exogenous sources of useable N may be relatively much more important than for other elements, since some meteorites and the comets are known to have a significant content of N-rich organics [140], including cyanides [141]. Cometary delivery of HCN has been shown be a major potential source of cyanide during individual impacts [129]. It has also been hypothesized, however, that, as impacts proceed, they will re-liberate significant amounts of regolith nitrate and result in intermediate, quasi-steady state concentrations for N (soil and atmosphere) [142]

Early Mars had more N_2_, since it can be lost by atmospheric escape and fixation into soil. Estimates based on isotopic composition and various loss mechanisms range from 13× [143] to up to 200× [144] higher concentrations than current levels. In comparison, early Earth’s N_2_ may have been only 2× or perhaps even less than the modern value [145]. Taking these ranges into account allows for early Mars original pN_2_ to have been as high as ~3% that of Earth’s.

Oxygen. In most planetary atmospheres, O_2_ is extremely low (with Earth as an exception because of its biosphere’s abundance of oxygenic photosynthesizing organisms). Oxygen is a key atom in the biochemistry of life, with one O atom for every two C atoms in the organic makeup of the microbial cell, about twice as many as N atoms [130]. The O in carboxylic acids, esters, sugars, phosphates, and a myriad of other organic molecules needed for the OoL is readily derived by reaction with the hydroxyl radical from the water medium and from the photochemical processing of the relatively abundant CO_2_ in the atmosphere and its soluble byproducts in the aqueous phase. Although the present-day Martian atmosphere has very low O_2_ abundances, recent modeling suggests that Mars may have had multiple, cyclical episodes of higher atmospheric O_2_ (e.g., ~10 mb) in the Noachian [146], which may have provided both challenges and opportunities for macrobiont chemical evolution. Indeed, carboxyl- and carbonyl-rich macromolecular organic carbon compounds have been described from three Martian meteorites so far [137]

Phosphorus. It was learned early that phosphorous could be extracted from Martian shergottite meteorites with mild acidification [147]. Detailed experiments on a variety of Mars-relevant P-containing minerals (merrillite, whitlockite, and chlorapatite) show significantly increased dissolution rates compared to the terrestrially more common fluorapatites, as well as the strong effect of promotion of solubilization by acidification [148].

From the Mars rover missions, mobile phosphorous was indicated at the Independence outcrop [149]. The numerous Wishstone and Watchtower rocks on Husband Hill have very high concentrations of phosphorus (>5 wt%, as P_2_O_5_-equivalent [150,151], compared to the 0.4–0.7 wt% in Mars igneous rocks, such as Adirondack-class [152] and shergottites [125]).

Abundant nodules, some of which are phosphorus-, Mn-, Ca- and S-rich, have been discovered in the Ayton sample near the Groken drill site in Gale crater, with up to 18 wt% equivalent P_2_O_5_ [153], and could be much higher for a Mn-P phase (without CaSO_4_).

The Martian meteorite NWA 7034 is a basaltic breccia with clasts representing four distinct lithologies. One of these lithologies, termed “FTP” (enriched in Fe, Ti, and P), has a P_2_O_5_ abundance range of 6 to 12 wt% (1-sigma), as chlorapatite [154]. This “Black Beauty” meteorite is thought to be representative of average Martian crust and global soil because of its bulk element composition, although it does not contain the high S, Cl, and Zn of Martian soil. It also contains clasts enriched in Mn^4+^ oxides, which have 2.4 wt% P_2_O_5_, indicative of aqueous alteration [155].

The abundant iron-nickel meteorites on the surface of Mars [156,157,158] could also become a source of utilizable P from their content of the mineral schreibersite, (Fe,Ni)_3_P [159,160]. The minimum abundance from the Curiosity transects is 800/km^2^ [158], which implies ~10^11^ irons on the surface of Mars. It also has been shown that, if ammonia solution is present, the readily soluble amidophosphate can be formed and would be an appropriate P-source for PCE [160]. The higher Ni in Martian soil may be an indicator for micro-particulates with this composition.

Given that early Mars and Earth could have had significant cyanide [12,36], as assumed by many hypotheses for the OoL, it has been shown that ferrocyanide or MgSO_4_ plus Na cyanide can enable the formation of organophosphates from hydroxyapatite [161].

Sulfur. Mars is clearly a sulfur-rich planet, compared to Earth [162,163]. This may be especially important because, as pointed out above, so many concepts for the early prebiotic chemical pathways make significant and often essential use of organic molecules containing sulfur atoms and/or inorganic compounds or minerals involving sulfur. This is the case for the cyanosulfidic pathways of the Sutherland group [35,109,164,165,166], the thioesters of de Duve [19], the Fe-S World of Wächtershäuser [25,26], the SO_2_ sequestration of HCHO of the Benner group [48], the cysteine primordial precursor of the Powner group [37], the sulfides for the hydrothermal vents [91,167], etc. On an early, wet, reducing and more sulfur-rich Mars, thiol-containing organics may have been far more prevalent than on early Earth, leading to sulfur organic chemistry in a variety of surface and subsurface environments and providing widespread cyanosulfidic chemistry and/or thioester chemical energy for a Martian origin of life.

Martian basalts, as evidenced by the shergottites and other meteorites, generally contain S (as sulfides) at a level [125] which is higher by about an order of magnitude than the composition of MORB basalts [168], and the global soils are enriched by a factor of more than an order of magnitude over the shergottites, with S equivalent to 4 to 8 wt% SO_3_ [123,124,169,170].

The Martian soils and sediments often have S as sulfates. Magnesium-rich sulfates are found in duricrusts [169], the Burns formation [171], soil trenches [172], evaporites in Gale crater [173], and Murray formation [174]. Orbital observations of Mg sulfates in both monohydrated (kieserite) and polyhydrated states have been discovered in many deposits [5]. MgSO_4_ is highly soluble, which can provide the Mg^2+^ cations used in many prebiotic chemical sequences, including the RNA world. Some microbes not only tolerate but grow well under 2 M MgSO_4_ concentrations [175].

Extensive ferric sulfate soil horizons occur on Husband Hill and Home Plate [150,176]. Mg and Fe sulfates are also a common constituent of Murray formation rocks in Gale crater [150].

Less soluble than MgSO_4_ are minerals composed primarily of CaSO_4_, which were nevertheless once in solution, as evidenced by their widespread occurrences as veins in Gale Crater [6] and also in Columbia Hills at Gusev crater [177].

The source of the high S in Martian soil is generally posited to be via magmatic release into the atmosphere [162,178,179], variously as H_2_S, SO_2_, or SO_3_, depending on the oxygen fugacity of the source at the time of release. Once in the atmosphere, the H_2_S may be quickly oxidized, mediated by strong UV photochemistry [112,180]. The SO_x_ forms will yield the sulfurous and sulfuric acids once they interact with water (aerosol, or subaerial). Similarly, the HCl and Cl_2_ released from magma can also impart acidity to the soil. In spite of the global soil’s putative high content of these acidifying species, its pH was found by the Phoenix mission to be circumneutral, with a pH of 7.7 ± 0.1 [181], which indicates the alteration of mafic mineral grains or interaction with intrinsic carbonate [182] to provide neutralization by their mild alkalinity in solution.

Not only the widespread sulfates in soils and sediments, but also the presence of sulfites has been inferred for some samples, based on release of reduced S-containing volatile compounds during EGA analyses by the SAM instrument [183].

In view of the widespread incorporation of [FeS] clusters into various critical enzymes, a PCE path to their generation has been investigated, using environmental UV to photo-oxidize the Fe^2+^ that would be generally available in early planetary environments [165]. Early formation of protoferredoxins would be a major step toward establishing electron storage and transport chains for a variety of biochemical pathways. That Mars is more abundantly endowed with both Fe and S lends support for the potential rise and evolution of these Fe-S based functions.

Organic molecules. The search for organic molecules on Mars has been long and determined. Early exploration with the Viking mission concluded that organic compounds were at levels below about 1 ppb, and the detection of chlorobenzene was ascribed to contamination [184]. These low levels in spite of infall of carbonaceous meteorites and interplanetary dust particles, not to mention the demonstration of organic synthesis from atmospheric conditions under UV irradiation [185], were explained as being the result of the strong oxidation power of the Martian atmosphere, driven by photochemical reactions [109].

Organics discovered in numerous Martian meteorites are indicated to have been synthesized by electrochemical reduction in the amount of CO_2_ by exposure of magnetites, Fe-sulfides and brines [137].

Subsequently, the MSL mission’s organic analyzer SAM detected ~10 ppb of halogenated organic molecules in some samples [186]. Additionally, still later, aromatics, aliphatics, thiophenes and other S-C compounds were detected at up to ~20 ppm C in mudstone samples from the Murray formation in Gale crater [187]. The concentrations of organics (released at high temperatures >600 °C) in Martian meteorites are 8 to 14 ppm in Tissint [188], indicating that indigenous abiotically synthesized organic materials provide a pool of building blocks for prebiotic reactions on Mars. For our example of 1:1 W/R ratio in mud, these would be a concentration of a factor of three or so less than 1 nM, even if all organics were soluble. Various concentration mechanisms envisioned in macrobiont scenarios could enable PCE to proceed.

Although the Viking GCMS had the demonstrated capability for detecting thiophenes as well as other organics [184], it did not detect such levels in any of the samples at Utopia Planitia or Chryse Planitia. This was apparently due to several differences for Viking analyses: only global soil samples could be acquired, rather than lithified sediments which may have been much better protected against atmospheric oxidation and ionizing radiation (GCR); the maximum pyrolysis temperature was 500 °C, whereas the organics detected by SAM were only released above this temperature; the sample size was smaller in mass and flash heated then held at maximum temperature for only 30 s, compared to a 10× larger sample and much slower temperature ramp for SAM, which resulted in several hours at temperatures of 500 °C to 820 °C; and, finally, a cost descope eliminated the direct-inlet to the Viking mass spectrometer, requiring the evolved volatiles to pass through the GC column before injection into the MS.

The contribution of accreted meteoritic matter to the regolith places a floor on organic matter that would be expected, independent of abiotic or biotic synthesis, and assuming no subsequent oxidative destruction. The nickel contents of the global Martian soils cluster around 450 ppm Ni [123,124], but Ni in the source igneous rocks is much lower (average of ~100 ppm for SNC meteorites [125] and MER igneous rocks in Gusev crater). If this excess of 350 ppm is solely due to meteoritic contributions, it implies an upper limit of 1000 ppm for exogenous carbon because the ratio of C/Ni (wt/wt) in CI meteorites and Tagish Lake is 3.1 [189]. This is, of course, far higher than the levels of organics detected so far on Mars. Not all meteorites which can contribute Ni are carbonaceous, but it is expected that the organic-rich versions would be more plentiful at the orbit of Mars and also would be more readily incorporated into soil because of their relative fragility and susceptibility to disintegrative weathering than the irons or other non-carbonaceous meteorites.

Clay minerals. Alteration of feldspars can lead to montmorillonite minerals. These have been detected at many locations on Mars by orbital spectroscopy, including other smectite clays of Mg/Fe varieties [5] as well as kaolinite, chlorite and illite clays, plus hydrated silica [190]. However, orbital observations are limited in spatial resolution and discrimination ability, such that additional discoveries of montmorillonite geochemistry that could not be detected from orbit have also been made in the Independence outcrop on Husband Hill [149], in the Esperance boxwork [191] in the rim of Endeavour Crater [177], and in numerous mudstones and sandstones in Gale crater [6]. Although clays are generally not major hosts of bioavailable CHNOPS elements, they do have important capabilities for the physi- and chemisorption of elements and PCE molecules from solutions, as well as catalytic roles for oligomerization reactions [49,192].

#### 3.1.2. Transition Elements

The significance of an enrichment of an element over its primary abundance in its igneous mineral precursor is not just that a 10-fold increase in boron, copper, or other trace element drives key reactions 10× faster or more complete, or that some critical threshold has been crossed. Most importantly, any enrichment or any depletion in an element typically indicates that it has been subjected to aqueous dissolution and transport [193]. Hence, it becomes “bioavailable” at the location detected, or, if depleted, at potentially some other location where it has become even more concentrated by aqueous-mediated processes. Depletions as indicators of mobilization have been more difficult to ascertain, but many different cases of significantly reduced levels of Mg, Fe, Mn, Ni, or Cr, have been detected [6,7,149,150,170,171,177].

Less common non-aqueous processes of segregation, such as magmatic differentiation or eolian sorting, may be responsible for changes but, if not, then the departure from the normal range of concentrations from the primary sources is indicative of mobilization. Aqueous environments are what are needed for the OoL because they provide the means for key reactants to come together in the same medium, which is already a prerequisite for abiotic progression towards and sustenance of LAWKI

In Gale crater, Stimson formation sandstones have been extensively leached of Mg, Al, Mn, Fe, Ni, and Zn [194] from their original mineral phases, as well as probably many other elements not detectable by the rover-based analytical systems.

Iron is ubiquitous on Mars. It has also already been demonstrated to occur in much more than a dozen different mineral forms, thanks to the Mössbauer instruments on the MER rovers [176], as well as remote sensing of minerals ranging from hematite to Fe-smectites. Although the oxidized, Fe^3+^ forms are more common, there are several mixed Fe^2+^Fe^3+^ minerals, as well as the Fe^2+^ in the primary igneous minerals olivine and pyroxene. Iron and nickel can also be available in their native forms (Fe° and Ni°) from the siderite meteorites which are surprisingly abundant at the surface of Mars [156,157].

**Figure 2:**
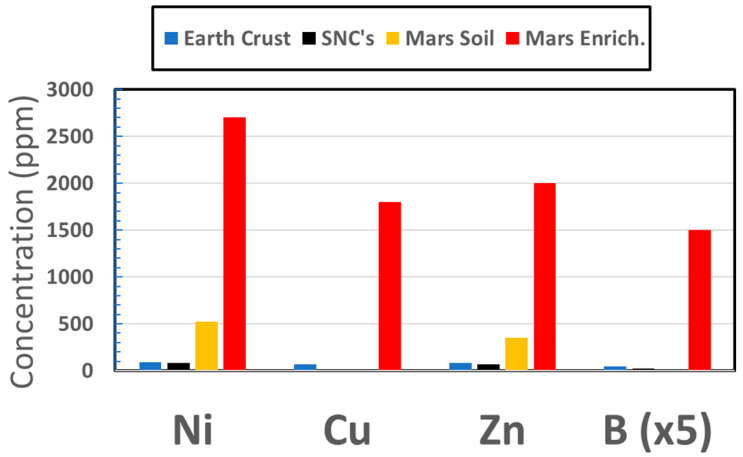
Examples of enriched occurrences of some key transition elements discovered during the MSL mission, compared to the Earth’s average crustal concentrations, SNC meteorites [125], and a typical Martian global soil composition [123]. “Mars enrichments”: nickel maxima (except for meteorites) for three rover missions (MER, MSL); Cu at Gale crater [196]; Zn also all three missions, plus up to 8000 ppm in Gale; boron at Gale [198].

Copper has been discovered at anomalously high concentrations at nearly a dozen locations in samples along the route of the Curiosity rover [195]. In the Kimberly formation, a concentration occurrence as high as 1100 ppm was found, some two orders of magnitude higher than for typical Martian meteorites of igneous composition [196] and much higher than typical crustal abundances on Earth; Figure 2. Copper enrichment at 580 ppm was also detected in target Liga at Gale [197], and a level of 230 ppm was discovered in the Independence outcrop [149] on Gusev crater’s Husband Hill. These data indicate that, not only had Cu been rendered mobile, but enrichments were relatively common, lending credibility to the possible occurrence on Mars of the Cu-catalyzed cyanosulfidic metabolic pathways to precursors for the three fundamental classes of biochemicals. Tracking of Cu abundances has shown that enhanced levels are found over a wide range of occurrences in Gale crater, especially in areas of phyllosilicate abundances [195].

Nickel and zinc have been routinely detected at surprising levels by the APXS (Alpha Particle X-ray Spectrometer) for more than a thousand measurements of soils, rocks, and sediments on three rover missions, with generally much higher abundances than for terrestrially analogous materials. These enrichments can be one or two orders of magnitude over terrestrial averages, Figure 2, and are only those which have been discovered inadvertently and generally without opportunities to further trace their origins.

Manganese. Enriched concentrations of Mn have been discovered repeatedly in Gale crater [199,200] and also in isolated occurrences at Endeavour crater [201]. These have been interpreted to implicate higher environmental oxidation potential and the presence of appreciable O_2_ in the past to produce MnO_2_ [200]. More recently, indigenous Mn oxides in the 4+ (oxidized) state have been identified in the Black Beauty meteorite pairs NWA 7034 and 7533, with the Mn-rich clasts containing up to 65 wt% MnO_2_ [155]. Additionally, MnO_2_ precipitation scavenges Zn and Ni, but not Cr [202], as seen in the Gale samples, and has led to the suggestion of the possible involvement of other oxidants, such as atmospheric agents (O_3_), nitrates or perchlorates, and an Eh above +500 mV for a pH~8 [202], whereas an Eh of about +300 mV was measured in the soil by the Phoenix mission [181]. These trace element correlations do not occur, however, for the high Mn-Mg-sulfate rock coatings discovered at Endeavour crater, which may indicate different conditions or mechanism(s), such as alternatives that have also been suggested for concentrating Mn on Mars [203].

Cobalt is very difficult to detect by APXS, because of obscuration of its K_α_ and K_β_ X-ray emissions by K_α_ lines of the much more abundant Fe and Ni. However, target Stephen in Gale crater provided a special opportunity, for which a Co concentration of 300 ppm was detected [197], a nearly tenfold enrichment over Shergotty cobalt [125].

Vanadium can vary over a range of roughly 50 to 500 ppm for various basalts and meteorites, with Shergottites at ~300 ppm [125]. In contemporary terrestrial soils, it can range widely from a few ppm to ~500 ppm [204]. At the higher levels, it would be enough for detection on Mars by X-ray fluorescence spectroscopy, except that two other elements which are normally at higher concentrations overlap too closely in emission energies for the accuracy of non-laboratory measurements (Ti K_β_ overlaps V K_α_, and Cr K_α_ overlaps V K_β_ X-ray emissions). However, if a Mars sample were enriched in V, while being lower in Ti than typical, a positive detection for V might be possible. The smaller analytical spot (~10^4^ times smaller area) of the PIXL XRF instrument on the Perseverance rover could in principle make such a determination if a “reduction spot” precipitate enriched in V, a potential chemical biosignature [205,206], were detected.

Tungsten is typically found at 0.1 to a maximum of 1 ppm in terrestrial basalts [207] and shergottites [125]. However, detecting W by remote XRF would be by its L_α_ emission, which is unfortunately sandwiched between the more common Ni K_β_ and Zn K_α_, both of which are typically at levels of hundreds of ppm in Mars soils.

Molybdenum is also present at ~1 ppm in basalts and meteorites, which is far too low for rover-based XRF detection. Additionally, its K_α_ emission will generally be obscured by the K_β_ from Zr, which typically occurs at one to two orders of magnitude higher concentrations. However, an oxide form of Mo, the molybdate ion MoO_4_^2−^, is soluble and could be mobilized and potentially detected if formed and sufficiently concentrated apart from minerals with nominal or lower concentrations of Zr.

For the V-W-Mo triumvirate, the most likely possible detection before samples are returned to Earth would be for V. These three elements often correlate in enrichments [204], such that detection of any one of these could be an indicator for the other two.

The elements As and Se can substitute for their corresponding higher row elements (P and S) in certain circumstances and seem to be essential elements for some organisms. However, they often are toxic and it is unknown if either may have had any important role in the early emergence of protometabolism.

#### 3.1.3. Other Key Elements

In addition to fundamental feedstock elements and catalytic ions, there are several elements which seem necessarily attendant to the origin of life because of their special properties and/or abundances. These include electrolyte elements, such as Na and K, accompanied by Cl, as well as stabilization elements, such as boron and certain divalent cations, such as Mg and Ca.

Electrolytes. Several ions inside cells are typically at far different concentrations from the medium they are in, as modulated by various controlling factors: ion channels which can be gated open or closed; active pumps which utilize chemical energy to transport ions against their concentration gradients; uptake and sequestration by organic constituents. Typically, for LAWKI, the element potassium is brought inside the cell, while Na and Cl are reduced relative to their concentrations in sea water. Another key element, Mg^2+^, is roughly at the same total concentration inside and outside, except that the large majority of the inside portion is bound up with ribosomes, ATP, proteins, and other macromolecules, such that its free concentration in the cytoplasm is greatly reduced [130].

Potassium. Wet–dry cycling yields for oligopeptide formation from glycine has been shown to be enhanced by as much as 10× when deliquescent salts are present, especially those of potassium phosphates, which also invoke a possible direct relevance of K to the OoL [208]. In its native igneous form, it is predominantly found in feldspars, although there can be occurrences in certain other minerals, such as the micas. In addition, its high-temperature polymorph, sanidine, has also been discovered in some samples on Mars [209]. Once K is released as the result of weathering, its various forms are quite soluble, providing great mobility and accessibility. However, it is readily adsorbed by minerals and organic matter. It is also a principal component of illite clay, which has been detected on Mars from orbit [5] and by Curiosity-based measurements in the Gale crater [210], and is often associated with other phyllosilicates, such as montmorillonite.

On Earth, K-feldspar is common in continental rocks, whereas it is minor or lacking in oceanic basalts, and the types of mafic and ultramafic assemblages that were prevalent prior to the formation of the continents. On Mars, K is at relatively low concentration in the global soil (0.5 wt% K_2_O), but occurs in several locations at higher concentrations (typically 0.25–0.5 wt%, rarely above 1.5%, but as high as 3.7 wt%, compared to Earth’s crustal average of 2.8 wt% and local values often much higher). Three different igneous polymorphs of K-feldspar have been found in Gale samples [6]. However, various other samples, such as Oudan, have no detectable crystalline K-feldspar but K_2_O is inferred at the level of 1.7 wt% in the amorphous material (which accounts for almost one-half of that sample) [6]. Similar amounts are inferred for the amorphous components of ordinary aeolian soils. K-bearing hydrated sulfate salts such as jarosite [5,176] and alunite [5] have also been discovered. At the Phoenix polar site, the measured concentration of K^+^ in aqueous solution with Martian soil [136] would be equivalent to about 10 mM for a 1:1 water/soil ratio.

Sodium is sometimes correlated with potassium in Mars samples, as well as with aluminum, which implicates feldspars as the actual, or original source. In a few cases, with higher values of Cl, the Na is correlated with that element, further indicating that Na^+^ ions were available in solution (although a major role in either biology or the PCE leading to an OoL is not typically attributed to chlorine). Positive ions are generally needed for charge balance, since many organics and the phosphates are negatively charged at neutral pH. There is also the need for osmotic balance of the intracellular fluid with respect to the extracellular medium.

Magnesium and calcium. The Group 2A elements of Mg and Ca are prominent bioinorganic chemicals [68], albeit with different functions. As widely acknowledged [69] and described previously, Mg^2+^ plays major roles in numerous biochemical and enzymatical processes of the key molecules of life. For example, at least five separate functions in ribosomal activity require Mg^2+^ [211] (although Fe^2+^ and Mn^2+^ can substitute for some of these functions [212]). Ca^2+^ also contributes too many important biological functions, although the ionic sizes and hence charge densities are quite different between these two cations [69] and lead to different utilizations. The salts of Mg are highly soluble, while Ca halides are also highly soluble but the sulfate is only sparingly soluble. Ca occurs in many primary minerals, especially the pyroxenes, plagioclase feldspars, and apatites, and is susceptible to release by aqueous alteration of these, with susceptibility generally in the order listed. Given the ubiquitous various occurrences of CaSO_4_ on Mars, Ca^2+^ has clearly been an available ion.

The global soil of Mars has a spectral signature evidencing ~2% MgCO_3_ [182]. Magnesite is poorly soluble in circumneutral H_2_O, but is readily solubilized by mild acids. A low water/rock ratio for a mix of global soil and water (wt/wt) could produce a high concentration of Mg^2+^, if all sulfate is present as soluble MgSO_4_. Although there is not yet direct X-ray diffraction evidence for MgSO_4_, such as kieserite or in higher hydrated states, strong correlations between Mg and S are observed in many locations, as cited above, while the S is known to be sulfate for a variety of reasons. For a typical SO_3_ concentration of 6 wt% in Martian global soil [123] and a water/rock ratio of 1:1, the Mg^2+^ from the equivalent of 9 wt% MgSO_4_ could reach as high as 750 mM, or about 15 times greater concentration than in Earth’s ocean waters. However, it is not clear that this amount of Mg is available from soil. The Phoenix polar mission measured soluble Mg and SO_3_ separately and concluded that a likely 2 wt% of soluble MgSO_4_ was present in soil in that area [213], inferring a Mg^2+^ concentration of 166 mM for our example W/R = 1, which is within the range of 83 to 185 mM, as derived from the Mg^2+^ concentration (within error bars, across different samples) measured. It is unknown whether the Phoenix soil has as high total SO_3_ as the typical global soil measured at six other mission locations (all in equatorial or mid-latitude locations). However, the early conclusion that the widespread Martian soil can supply large amounts of Mg^2+^, sulfate anion, and chlorine and oxychlorine species is secure. Whether this global soil that is universally available in the present epoch also had this same composition in the Noachian, prior to the theiikian interval [214] when abundant bedded sulfates were deposited, is not yet determined but would seem less likely. Nonetheless, that the Martian lithosphere is sulfur-rich compared to terrestrial soils seems incontrovertible. Hydrothermal processing of mafic rocks on Earth, including at the suboceanic vents, typically result in high concentrations of S, chiefly in the reduced form of metal sulfides. Our example of a 1:1 ratio for W/R is a mud, whereas a pond will allow soil particles to settle. The saturation concentration of MgSO_4_ is 2900 mM at +20 °C (2200 mM at 0 °C). Thus, if a pond leaches its bottoms and sides, as well as its foreshore and any airfall dust, the MgSO_4_ could rise to very high levels. This salt is also very hygroscopic and forms several high-order hydrates (e.g., epsomite at 7 H_2_O per MgSO_4_).

Muddy water, as opposed to a wet mud, could reduce the Mg^2+^ concentration to a greater extent. This could still easily be adequate to foster RNAzyme activity since the test-tube evolution experiments are successful when conducted at high Mg^2+^ levels [63], typically 50 to 200 mM [56,62]. Although cations accelerate the natural degradation of RNA in solution by hydrolytic cleavage, the Mg^2+^ catalyzes this less severely than Fe^2+^ or Mn^2+^, and also helps stabilize the three-dimensional conformations of RNA, while at pH < 5.4, increasing Mg^2+^ concentration to 50 mM actually slows down the degradative cleavage reaction [16].

Boron. At Gale crater up to 300 ppm boron has been detected [198,215], Figure 2, but the CCAM instrument uses laser ionization breakdown spectrometry (LIBS), which can detect B only in low-Fe samples, such as CaSO_4_ veins, because of interfering emission lines from the otherwise ubiquitous Fe on Mars. Using different techniques in the laboratory to analyze the Nakhla Martian meteorite, boron has been found to be enriched to levels of 160 ppm in alteration zones associated with Fe-rich smectite clay [216]. On Earth, this element can also be found enriched in hot springs [217].

#### 3.1.4. Elements Availability

Mars’ endowment with the elements of life is adequate to supply not only the nutrients for microbial LAWKI (i.e., habitability), but also the feedstocks and catalysts needed for an origin of life. The enrichments noted above confirm the extraction and concentration of key ingredients. For these ingredients to be available, however, they must have adequate solubility in aqueous media. The solubility product (K_sp_) for compounds of these elements is generally high, but depends on the valence state. Aside from the K_sp_ for pure H_2_O, there can be dependencies on other components, but the most important mitigating factors can be the pH and oxidation potential (Eh) within the aqueous medium. Thus, because much of the Fe on Mars is now in the Fe^3+^ form, its K_sp_ is extremely low, except for conditions where the very low pH and Eh portion of the relevant Pourbaix diagram is realized. However, it is the ferrous form that is catalytic and involved in (FeS) clusters. At the low Eh for early Earth, the expected concentration of Fe^2+^ in the ocean would be as much as four orders of magnitude higher than the Fe concentration today, while Co and Mn would also be higher; in contrast, the Cu, Mo, Zn, and Ni concentrations would be much lower [218]. Cycling between more oxidizing and more reducing atmospheric states, as recently proposed [146], could induce significant variations in relative ionic concentrations among the various redox-sensitive elements. Combined with the discovery of ferrous smectite in the Gale crater and laboratory oxidation experiments lends credence to the hypothesis that the Fe^3+^ smectites observed by orbital spectroscopic mapping were originally in the Fe^2+^ form before being altered further [219].

Since Mars is a sulfur-rich world in comparison to the surface of the Earth, when volcanic emissions of H_2_S and SO_2_ are converted to SO_3_ by photochemical byproducts [180,220,221], then Martian shallow aqueous reservoirs will have their pH lowered, resulting in the formation of sulfates [222] which are highly soluble and can provide high levels of availability for all relevant elements, except for Ca and Fe^3+^. Jarosite in the Burns formation has been cited as a clear indicator of significant acidity (pH ~3) at the time and the location where it was formed [171]. As basic environmental minerals react and drive the pH toward neutrality, new emissions can reverse the process in shallow lakes and ponds, to restore the higher levels of needed elements. However, such reactions require time, depending on the minerals available as well their grain size and armoring effects [178], providing the macrobiont with slowly varying pH which may facilitate some steps of PCE. Volcanic emissions of Cl_2_ and HCl can also produce chlorides that form highly soluble salts of these elements [162,223]. In the widespread Martian global soil, the S/Cl ratio (atom/atom) is ~4:1 [123,124].

**Figure 3:**
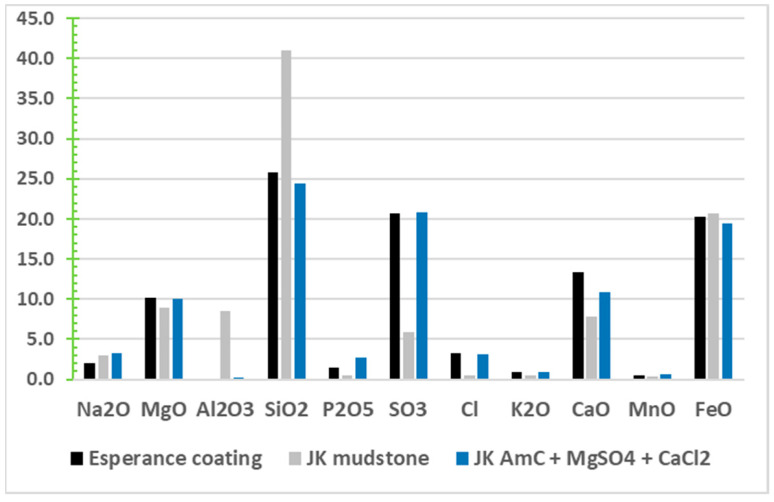
Evidence for similar amorphous material at widely separated sites: Esperance coating [191] at Endeavour crater compared to JohnKlein (JK) and its amorphous component [224] plus salts at Gale crater (85% JK AmC, 13% MgSO_4_, 2% CaCl_2_).

From the X-ray diffractograms of the CheMin instrument, most samples of soils and sediments at Gale have a significant component of X-ray amorphous material (15 to 70 wt%) [6,224]. By assuming elemental compositions of the clays, igneous silicates, and other minerals exhibiting diffraction peaks, the net elemental composition of the amorphous components (AmC) can be inferred from the APXS measurements of the bulk sample. The resulting compositions of AmC are extraordinarily disparate among samples (e.g., SiO_2_ at 29 to 75 wt%, FeO of 5 to 30 wt%, SO_3_ of 1 to 22 wt%, and Cl as high as 6 wt%). Amorphous material can include MgSO_4_, which has been widely detected by other means but not in crystalline form by CheMin. Given the non-consistent composition of this AmC material, and general lack of correlation between most elements, many elements must be individually mobilized, with relative concentrations resulting from various local conditions at the time of immobilization. This implies a wide range of element availability for Na, Mg, Si, P, S, Cl, K, Ca, and Fe. This phenomenon is not restricted to Gale crater. The dark coating of the Esperance montmorillonite-composition fracture fills [191] at Endeavour crater matches Gale’s amorphous material in the JohnKlein sample (a mudstone in the Yellowknife Bay formation [225] with 19% AmC) [224] for all major elements analyzed if that sample would simply have more MgSO_4_ and some MgCl_2_, as shown in Figure 3. Finding such similar amorphous materials in both a mudstone at Gale crater and, some 17,000 km distant, as a coating along the rim of Endeavour crater, implies that amorphous materials may be ubiquitous on Mars, and hence its elements would be widely available. It is noteworthy that only the MSL mission, with its CheMin diffractometer, has had the capability to detect and infer the composition of amorphous materials.

### 3.2. Energy Sources on Mars

At aphelion, Mars receives only a little more than one-third the solar flux of Earth, and at its perihelion still only one half, although these are more than adequate for photosynthesis. This factor of between 2× and 3× less sunlight, assuming a transparent atmosphere, is not a significant difference for the UV flux, and the diurnal cycle for the very early Earth was shorter from the closer proximity of the moon.

A variety of redox energy couples would have been available on early Mars [226,227], in addition to the solar sources of ultraviolet and visible energy. The reduction in the amount of CO_2_ to organics by H_2_ is an exergonic reaction and methanogenesis is considered a likely early metabolism. In a primitive syntrophic relationship, energetic reactions of metabolism could have also worked in the other direction to oxidize methane back to CO_2_ in analogy with methylatrophic catabolism with a suitable oxidizer.

If sulfates and nitrates were produced by photooxidation processes, there would be ample redox couples as electron acceptors with any H_2_ or CH_4_ in the atmosphere. Sulfur, having multiple oxidation states and with −2, 0, +2, +4 and +6 all being relatively stable, has numerous energy-releasing reaction pathways with end products ranging from H_2_S to native sulfur to sulfate, and various microbes that can take advantage of these transitions to drive their metabolic functions.

The transition metal elements, with their partially filled d orbitals, can occur in two or more oxidation states, providing well-known couples with Fe and Mn. Nitrate-utilizing microbes can combine that electron acceptor with Fe^2+^ as the electron donor as one energetic pathway analogous to the metabolism of a variety of iron-oxidizing microbes on Earth [228]. Iron meteorite plus an oxygen source has been shown to provide an ample source of energy for metabolism and the growth of acidophilic chemolithoautotrophic microorganisms [229].

Given that hydrothermal processes are available, due to local volcanic activity or deep-seated thermal transients induced by the conversion of kinetic energy of the larger hypervelocity impactors, there could be an even larger range of potential redox couples [81].

### 3.3. Settings on Mars

The geologic processes of Mars are far from fully understood [230], although many of the igneous and sedimentary features have analogs that are well known on Earth. Unlike Earth, much of the earliest geology of Mars is preserved in the intercrater regions. Far less known and understood are the early atmospheric and hydrologic environments, and it has been challenging for climate modelers to find parameter sets that make plausible the temperatures that would have been needed for the availability of liquid water, rather than ice, to form the geomorphic modifications [4,114] and aqueous-mediated geochemical concentrations [5] that are widespread across the planet.

#### 3.3.1. Early Mars as Compared to Early Earth

Early Earth was very wet, with a possibly globe-encircling ocean and little if any exposed land other than the summits of island arc volcanoes and micro-continents [231]. This scenario is based on the modeled slow emergence of plate tectonics, from which the continents were formed, although the timing of the rise of the continents remains uncertain and highly controversial, with recent evidence from zircon trace elements of the existence of felsic crust within the first 500 Myr of Earth’s history [232]. For all the OoL hypotheses requiring land and subaerial exposures, the expectations for a successful origin would be constrained if Earth’s ocean were global and tectonic activity subdued [233].

For wet–dry cycling to be possible, the planetary body would need exposed land as well as shallow ponds, lakes, and seashores [234]. Mars could be far more favorable in terms of the amount of exposed land because even the most optimistic estimate of the size of an ocean in the lowlands of the northern hemisphere is less than one-third of the total surface area of Mars and would require a global equivalent layer (GEL) of ~550 m of H_2_O [111]. This is at the high end compared to estimates that, by the late Noachian, there was only approximately one-tenth of this amount of water available, and which would, therefore, prevent fully filled ocean-sized bodies of water [235].

If, for example, 2% of the surface of the early Earth were exposed volcanic land masses, and 30% of Mars was submerged, then Mars would have 10× more subaerial land to enable the advantages of factors such as wet–dry cycling, UV irradiation, atmospheric stimulation, concentration of ingredients, etc. If only 15% of Mars were submerged and 25% of Earth was land, the planets would have equal amounts of subaerial terrain. However, with what is perhaps a more likely situation at early times (5% Mars submerged and 95% of Earth submerged) Mars would still have a 3× greater exposure of land in spite of being a smaller planet.

Punctuated Climate. Detailed climate models currently indicate that even with a supply of H_2_ gas to the primitive atmosphere, the pressure-broadening of absorption lines for a CO_2_ greenhouse effect [112,130] is insufficient to maintain a perennially warm climate to prevent widespread freezing [4,114,120,236].

Although methane is also a potent greenhouse gas, and would be a welcome addition as a feedstock for PCE, its photochemical lifetime would be short. Furthermore, its production attendant with the serpentinization reaction would be low compared to hydrogen [110].

Although there is no evidence of the quantity of H_2_ that would have been present on Earth in its Hadean eon, or on early Mars in its early Hesperian phase, or even definitive evidence of its presence at all, there are many possible sources of H_2_ that lend credence to its likely contribution as the key component for more tightly closing the early strong greenhouses on both planets. In addition to the hydrothermal serpentinization reaction, dihydrogen can be produced by several processes, including magmatic devolatilization; radiolysis of H_2_O by ionizing radiation from mineral K, U, and Th; and H_2_O reaction with dangling bonds or radicals on fresh mineral surfaces formed by rock abrasion and fractures [116]. Magnetite is ubiquitous on Mars [237] and the direct reaction of magnetite with H_2_O has been shown to provide yet another de novo source of H_2_ to enable a warmer early Mars [238].

Models of the effects during the creation of even the ~5000 “medium-sized” craters (>30 km diameter) on Mars [239] predict craters of this size and larger can produce major transient warm periods due to the greenhouse effects of the release of CO_2_ and H_2_O from the target material, and which can last from months to decades and centuries [240], but not sufficiently for longer-term warming [241]. Some models question whether such events are sufficient to cause the formation of the valley networks because the global effects are too short-lived [236]. However, other models conclude that 100 km impactors could create enough H_2_ and heat to raise a cold Noachian temperature to above melting for millions of years [242] and recent models suggest ample temporary climate change to produce the valley networks and other fluvial as well as lacustrine features that have been observed [146].

Irrespective of models, numerous examples of diagenetic episodes of aqueous alteration have been discovered in Gale crater, including potassic sandstone in the Kimberley area [243] and sediments in the Vera Rubin Ridge (formerly “hematite ridge”) [244,245]. Compositions of various diverse mudstones in Gale crater, combined with the inspection of sedimentary relationships, have led to a model of alkaline fluids in the Yellowknife Bay area (Mg-rich concretions), acidic fluids in Pahrump Hills (jarosite, and mobility of Zn, Ni, Mn, Mg, Ni, and S), hydrothermal fluids at Ouudam (gray hematite, opal-CT), high-redox, S-rich fluids elsewhere, as well as CaSO_4_ fracture-fills crosscutting earlier diagenetic features [246]. Each observed diagenetic episode can be the result of numerous wetting, dry-out, and re-wetting events. The local environments for lithified sediments most relevant to biotic evolution may be where there are subaerial exposures.

Estimates of the effectiveness of splash erosion on Mars is used to infer widespread rainfall for the formation of the valley networks, once infiltration losses could be minimized by the fine particulates from clay formation [247]. An examination of 13 open- and closed-basin lakes resulted in estimates for precipitation minimums (rain and snowfall) of 4 to 159 m for their catchment-averaged runoff [248]. However, differing models suggest that rainfall on Mars may have been much rarer than on Earth [249]. Snowfall and/or cold trapping of H_2_O vapor as ice could build reservoirs which, during warmer intervals, could be melted to produce runoff for the formation of valley networks.

**Figure 4:**
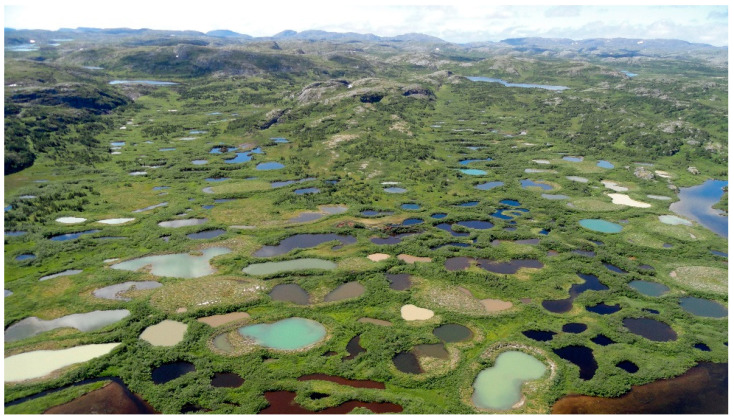
Diverse thermokarst ponds in Sheldrake River valley near Nunavik, Quebec in the Canadian subarctic (56° N) [253]. Photo-credit, J. Comte (Institut national de la recherche and Centre for Northern Studies).

Geomorphological analyses of the Kasei Valles region indicate episodic flooding with at least five periods of channel flows during 3.7 to 2 Ga, evidencing an active hydrological cycle well into the Amazonian [250]. Features implying thermokarst lakes and ponds also dating from the late Amazonian have been observed in Utopia and Elysium Planitiae [251]. Fan units of Amazonian age in Gale crater provide evidence of surface flow [252]. An example of extreme formation of diverse ponds in warming permafrost areas on Earth is seen in Figure 4.

**Figure 5:**
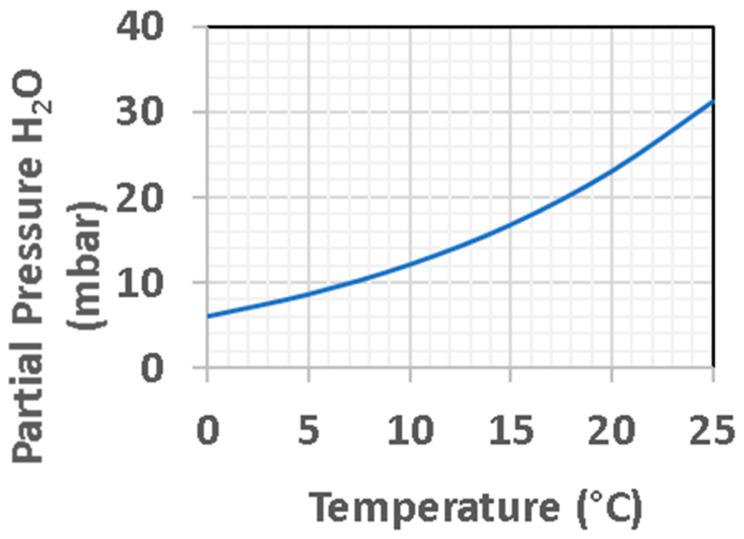
Partial pressure of pure H_2_O, setting the limit on boiling (brines will be stable to higher temperatures).

Cold temperatures. Although there is great emphasis and interest in hydrothermal regimes, a case has also been made for a cold OoL [106]. A cool or cold early Mars has often been predicted [114,146,254]. As seen in Figure 5, if the predominant temperature for exposed bodies of H_2_O were +20 °C maximum (southern summer), and only ~1% of the estimated inventory of buried CO_2_ (as carbonates) were released, it could provide an atmospheric pressure of 23 mbar to prevent H_2_O from boiling (only a three-fold increase over the present 6 mbar). Thus, an initial Martian atmosphere at 2 bars total pressure could decay by a factor of ~100× due to carbonate formation and escape to space, while still all the time enabling Martian H_2_O to be liquid in specific locations without excessive loss rates and dispersal due to boiling.

Alternatively, the average temperature could be much lower but surface patches with pro-solar slopes, modest-to-low albedo, and low thermal inertia could be heated beyond the ice melting point and perhaps up to +20 °C peak temperature during daytime. Repeated transient cycles of wetting and drying can be especially advantageous to PCE formation of polymers. Due to obliquity cycling, and an analysis which finds that only 500,000 years ago Mars was at its lowest obliquity and with a predicted rise in atmospheric pressure to 31 mb [255], it might be possible that OoL processes could be ongoing in the most recent epoch.

**Figure 6:**
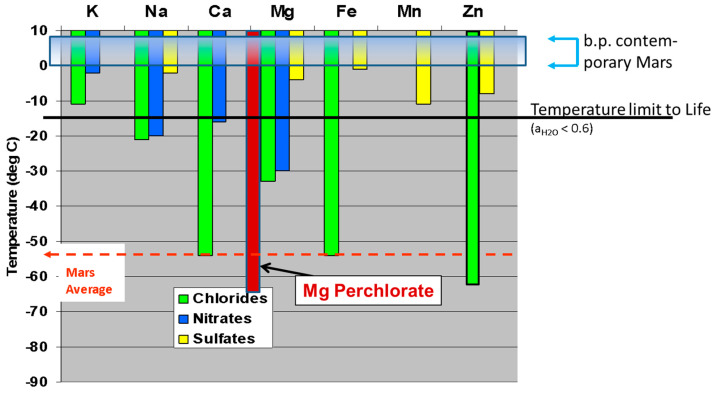
Freezing-point depression for salty brines, which extend the mobility of aqueous media in a sub-zero environment, but do not fully extend the temperature range for metabolic functionalities.

If brines are formed, especially those containing halides, the liquid regime is extended to yet lower temperatures. This implies higher ionic strength in the milieu in which prebiotic syntheses and processes must occur. In Figure 6, some candidate salts on Mars which can depress the freezing point of their brines through formation of eutectics are shown. Sulfates are poor performers, but chlorides and the oxychlorines readily block entry into the solid state. These brine media also have greatly increased viscosity, which slows diffusion rates and hence promotes the spatial heterogeneities in a pond that can aid the semi-sequestered development of different key functions needed for the comprehensive set of proto-metabolic activities of life forms (nutrient acquisition, component synthesis, energy management, waste management).

Occasional traces of chloride enrichments by rover instruments, especially on rock surfaces [256], have given way to more numerous detections, including hundreds from orbit [223,257]. In situ investigations in Gale crater [258] tend to indicate Na as the chloride salt. Even when the a_w_ at highly depressed freezing points may be too low for cellular growth and reproduction, the frigid environment might provide a unique and favorable intermediate environment for some processes of PCE.

Although there are psychrophilic organisms which can conduct metabolic activities and reproduce at temperatures somewhat below 0 °C (to about −10 or −15 °C) [259], some prebiotic reaction pathways may actually be strengthened or enabled by cycling above and below freezing, i.e., the freeze–thaw process analogous to wet–dry cycling [102,103,104,105,106].

#### 3.3.2. Subaerial Terrain Proto-Macrobionts

Mars has abundant locales on its surface which could host the origin of life. These include both ambient temperature regimes and hydrothermal settings.

Ambient settings. In addition to the natural undulations of surface topography due to endogenic processes, there is the exogenous influence that creates abundant basins in the earliest history of planets, i.e., the terminal accretion phase which leaves the scars of impact craters as evidence of its progression.

From a study of world occurrences [260], there are ~250 million ponds and lakes with equivalent diameter of 30 m or larger on the present surface of the Earth.

The number of craters on Mars >1 km diameter, is a minimum of 380,000 [239,261] (by actual counts, but this does not include craters modified beyond recognition after their original formation). From the fitted slope exponent of −1.46 for the cumulative size distribution at the lowest sizes, it is projected that there could have been a minimum of 50 million primary craters greater than 30 m in diameter, and perhaps ten times this many craters due to secondaries created by larger primary impactors (fitted exponent of −2.48). In a separate analysis, focused just on the Meridiani Planum area [262], the measured number of craters >30 m in diameter is 1.1 per km^2^, which extrapolates to over 150 million craters of this size or larger on Mars, with 90% of those being between 30 and 300 m in diameter. These estimates are of the same order of magnitude as the number of ponds this size or larger on Earth today.

Unlike impact craters on Earth, these observed Martian craters have survived >3 Gyr’s of geologic history since their formation. Craters smaller than 1 km will not have as significant heat energy density (J/kg) imparted to their vicinity, since they are evidence of a lesser deposition of kinetic energy, but they do form natural depressions for ponds of diameter up to the size of their rims. Other geologic forces will create additional natural basins in the remaining intercrater terrains.

The number of potential subaerial ponds is, therefore, extremely large on both planets, thereby facilitating the possibility of an OoL. However, they must be supplied with water to be effective as proto-macrobiont settings. On a very wet planet such as Earth, the large majority will be wet, or submerged beneath the ocean, whereas on Mars that essential condition is a function of not just location but also of climates and geologic time.

The Martian climate is now too cold to avoid the freezing of even large, exposed bodies of water. In spite of the faint early sun, however, the greenhouse was sufficiently effective that valley networks could be carved and perhaps even major bodies of water could form [111,263]. Topographic analyses of geomorphologic features indicate flooding of the large plains in the northern hemisphere to create a small ocean, followed (in time) by a smaller sea and accompanied by episodic occurrences of distributed lakes [263], shallow sediments [173], and thereby, by extension, of ponds. These major inundations span the Noachian to the late Hesperian, the end of which, ~3.7 Ga, coincides with the range of evidence that the establishment of a biosphere had already begun on Earth [264]. Lakes fed and discharged by the valley systems were comparable in volumes of H_2_O to the small seas on Earth [265], although many might have been short-lived, judging by the general lack of detectable chemical alteration products by orbital spectroscopy [266].

From MSL’s in situ exploration, abundant evidence of past activity of liquid water includes alteration chemistries (clays, salts) [6,267]. The Mg-Fe carbonate (Comanche outcrop) in Columbia Hills of Gusev, a former crater lake, is evidence of the ephemeral, mostly low-temperature alteration of mafic rocks [268].

The history of Vera Rubin Ridge at Gale crater indicates multiple episodes of groundwater interactions [269], evidenced in part by elevated concentrations of Mn. Accompanying long-term episodes of aqueous activity would have been undoubtedly shorter durations of superposed fluctuations which enhance the opportunities for concentration/dilution events and wet–dry cycles. Alternating episodes of wet–dry environments have been implicated by the chemostratigraphy of Mt. Sharp [270].

Although Gale itself is ancient, and its sedimentary load formed during the Hesperian, the measured low ^36^Ar abundance also suggests that water–rock interactions continued to occur well into the Amazonian [271]. Stratigraphic sections observed from orbit indicated enrichments in hematite, phyllosilicate, and sulfates, which implied extensive aqueous alteration [272]. Occurrences of phyllosilicates, sulfates (Mg, Ca), as well as sulfate-independent Mg enrichments at differing concentrations among individual samples taken by the Curiosity rover, show that environments were dynamic on a small-scale [6]. The indications of a redox-stratified lake with iron precipitates is consistent with magnetite levels progressing to hematite at higher stratigraphic levels [273], and is indicative of variable redox conditions which could support a diverse community of chemolithoautotrophs, as in terrestrial redox-stratified lakes [274].

**Figure 7:**
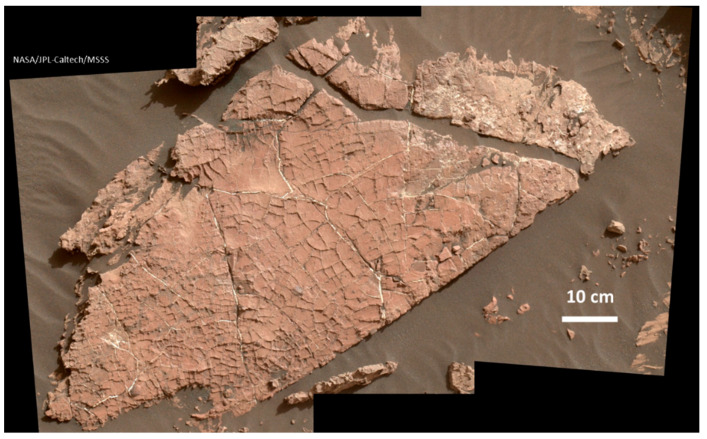
Mudcrack pattern of the “Old Soaker” slab (Sutton Island member of Murray Formation), a red mudstone overlying gray sandstone as imaged by the primary camera of the MSL Curiosity rover, sol 1555) [275].

Foreshore mudcracks. A potential feature of special interest for the OoL is mudcrack patterns [13]. On Mars, there has so far been discovered a clear example of an area of mudcracks, which is indicative of repeated wet–dry cycling along the shoreline of an oscillating lake level [275], as seen in Figure 7 of the “Old Soaker” unit.

Although this may seem rare, the sum total of Martian terrain that has been imaged so far by the four landers and five rovers at the minimum resolution needed to detect such patterns (3 mm), amounts to only 3 × 10^−8^ of the total surface area of the planet. Thus, there could be tens of millions of mudcrack units on Mars that have not yet been imaged but may have provided the heterogenous environmental conditions conducive to prebiotic chemical evolution [13]. Additionally, some mudcrack patterns will have become filled in by eolian-mobilized dust fallout and surface saltation or degraded beyond recognition by eolian abrasion. It is hypothesized that Gale crater was often wet, but on the basis of the presence of highly soluble perchlorate, there were dry periods that extended well into the Amazonian Period [276].

Hot springs hydrothermal activity. In spite of the presence of giant shield volcanoes on Mars, a tally of features nonetheless concludes there has been only a fraction (~2%) of the cumulative magmatic extrusive volumetric activity on Earth [277]. As another source of thermal energy, the buried heat from each hypervelocity impact on Mars, could also generate hydrothermal activity if H_2_O were available (e.g., as permafrost ice) [96,278].

A plethora of geochemical evidence of localized aqueous activity has come from the in situ exploration in Gusev and Gale craters. At Home Plate in Columbia Hills, there is high SiO_2_ with morphologic evidence for silica sinter similar to that at the hot springs at El Tatio, Chile [279]. High silica (~90% opal-A) enrichments have also been detected at Gale crater [280]. From orbit, it is difficult to detect the silica deposits expected from hot springs [281], but several light-toned deposits in Valles Marineris, the chaotic terrain, and some large craters has been interpreted as indicators for large-scale spring deposits [282].

A highly salt-enriched soil at Paso Robles on Husband Hill has been interpreted to have a hydrothermal origin [170]. In the Kimberly formation at Gale crater, there are occurrences of sanidine, the high temperature polymorph of K-feldspar that forms above 100 °C, which is evidence for hydrothermal activity similar to occurrences in the summit areas of Maunakea volcano in Hawaii [204]. Other minerals, such as tridymite, provide additional evidence of a hydrothermal history at Gale [194] and there is also evidence from the high concentrations of Ge and Zn detected in some sedimentary rocks [283].

It has been argued that subsurface hydrothermal activities were “abundant” on Mars as potential locales for the OoL, based on nearly three hundred exhumed sites detected by orbital remote sensing of relevant compositions (silica, carbonate, serpentine, and certain clays) which could be indicative of high-temperature geochemical alteration [284].

Evidence also comes from Martian meteorites, including the nakhlites, wherein phyllosilicates and Fe-rich carbonates were formed by high temperature processes [285]. Within the Tissint Martian meteorite are features that have been described as miniature vent-like morphologies and contain anhydrite, pyrrhotite, and magnetite nanophases with montmorillonite and associated organic nitrogen and oxygen compounds [185]. These features are in the 10 s of microns size range with obvious redox conditions existing on the scale of only tens of nanometers. If ubiquitous on Mars, their aggregate opportunities could provide significant opportunities for PCE.

#### 3.3.3. Suboceanic Hydrothermal Proto-Macrobionts

A spectroscopic analog to the Lost City hydrothermal field is claimed for the Nili Fossae region on Mars [286], based on the occurrence of Mg-rich serpentine, Ca carbonates, talc, and amphiboles.

Many observations of mineralogy from Mars orbit have been interpreted as indicating individual areas of former hydrothermal activity [287,288,289,290], including the Eridania region which may even have once been an undersea setting [291].

Given the smaller size and possibly shorter lifetime of one or more oceans on Mars [111,250,263], coupled with the lower level of volcanic activity [277] and apparent lack of tectonic plate activity on Mars [292], the inferred likelihood of an origin of life by the pathway of oceanic hydrothermal vents must be much lower than for Earth.

## 4. Discussion

Because we have a minimum date for the OoL on Earth [264,293], we can compare the array of suitable settings on Mars and Earth in those earliest times to gauge whether it is reasonable to expect that life could or should have also arisen on Mars.

### 4.1. When Would Be an OoL on Mars: Past, Present, Future

Based on the early appearance of life on planet Earth, the likelihood that life also arose on Mars could be high since both planets had liquid water coexisting with similar basaltic surfaces and reduced greenhouse gases. After the cessation of extremely adverse conditions at the time of the formation of the Earth and its Moon, appropriate environments conducive to the formation of suitable settings, i.e., proto-macrobionts [13], there would elapse periods until the rise of the first life forms, which could accomplish wide-spread colonization. From age dating relative to the formation of refractory material (calcium aluminum inclusions) and from accretion models, it can be inferred that the moon-forming Theia impactor occurred at 20 to 100 Myr after the formation of the Earth itself [294], giving Mars that first interval of time as a “head start” for an origin of life since the moon formation event would have destroyed any early PCE or OoL. Although the Theia impact created a molten silicate surface, it would have cooled very rapidly (~ kyr) down to a 100 °C temperature.

During the decay of the heavy bombardment, the earliest time an OoL could have begun on Earth is variously estimated somewhere between 4.5 and 3.9 Ga [264]. However, evidence for a biosphere-scale abundance of life is by ~3.7 Ga [264,293], or perhaps even earlier [295]. This implies about 500 ± 300 Myr for the progression from a lifeless planet to one that is widely inhabited. A case has also been made for life to have arisen during an even shorter interval of ~100 Myr [295], following the last globally sterilizing large impact [97]. From Mars’ location and size, its final global-scale sterilizing event by some giant impactor could have been even earlier than for Earth.

However, many investigators have pointed out that the duration for forming a living organism in any given setting could be much shorter, perhaps on the scale of a thousand years, or even less. This is because an assemblage of organic molecules and activities incipient to metabolism and RNA propagation will tend toward chaos, degradation, and decay unless a Darwinian evolutionary advantage is established. Once genetic properties become established, progression toward competent cellular life can be much more rapid. This is important because environmental settings can change on short time scales, and suitable conditions must persist for a sufficiently time for the complex sequence of PCE events to occur and to transition to entities that are alive before conditions become locally unsuitable.

Even if favorable climates were shorter on Mars, they could have been more than adequate because the OoL may be a rapid process for any given proto-macrobiont.

Nature changes on all time scales. A Martian lake that reaches the overflow or a breakout point may take years or decades to build up its inventory of brine, whether by runoff of precipitation or melting of cold-trapped ice. Once the discharge begins, however, it may complete its course in only hours or a few days [263].

A light photon emitted from the solar corona will take 8.3 min to reach the Earth (or 12.6 min to Mars), yet interact with its target atom, molecule or crystal lattice to release its energy to cause fundamental electrochemical changes in times measured in just nanoseconds. Geologic eons and eras are measured in millions of years, yet geysers erupt and subside in minutes.

How fast could be the rise of reproductive entities in a favorable proto-macrobiont setting? Some bacterial cells can fully reproduce themselves in less than 20 min [130] (primitive RNAzymes can replicate even faster). This biological feat requires the new synthesis of thousands of different molecules, made possible only because thousands of genes, proteins, and ribosomes are working in parallel and catalyzing reactions at high speeds [130]. In a prebiotic environment, the chemistries may also be complex, but are not coordinated or regulated, except to the degree that some molecules are autocatalytic for their own synthesis from available precursor molecules. Once a replicating system with heredity does form, such as an RNAzyme, the replication of programmed molecules can transition to exponential increases in rates of manufacture of useful products and improvements.

Furthermore, the natural lifetimes of most suggested settings for the OoL are not necessarily long compared to geological time scales of millions of years. How long can a pond avoid losing its water in any extended period of dry weather? How long can a given chimney formation of a hydrothermal vent remain active before it loses its connection with source water or becomes clogged due to excessive precipitation? How long can a hot spring continue to be hot? For planet Earth, virtually all the settings posited for macrobiont status are susceptible to having short lifetimes compared to geological time itself. Local activities are generally more realistically scaled as decades or centuries, but seldom multi-millennia. For planet Mars, these may be much longer, given its lower levels of almost all types of geologic activity [277].

Given that life on Earth did clearly start very early, and that early Mars was just as clement, if not more so, there is no justification in concluding that an OoL is a low-probability event. At this time, there is still no clear path susceptible to the de novo calculation of the likelihood for an origin of life on either planet, due to our still-nascent understanding of all the viable pathways by which prebiotic chemical evolution could occur, in addition to also not understanding all the suitable local environments that may have been abundant in those early millennia. These gaps in knowledge, especially for the former, have previously led to the general conclusion that the OoL may be a low-probability event.

An origin of life on small planetary bodies, such as the moons and minor planets of our solar system, is extremely unlikely for LAWKI because of a lack of a sustainable liquid form for H_2_O, except for the case of tidally pumped but ice-capped H_2_O-rich moons close to their host planet. For subaerial macrobionts, the planetary body must be large enough that its gravity sustains an atmospheric pressure greater than 6 mb. It is important to recognize that at these planetary scales, thousands of OHV’s or millions of lakes can be hosted, and, therefore, the likelihood of one or more of these settings becoming transformed to a macrobiont can overcome the low probability of any single setting becoming so, especially considering the magnitude of geologic time over which various PCE’s would have the opportunity to reach fruition.

It is clear that, in the mid-Noachian, there were abundant opportunities for the rise of life on Mars. Given the evidence of a slow progression of climate conditions toward those of the Hesperian Period [296], there was additional abundant time for the OoL, although the episodic freeze–thaw climate cycling envisioned by various groups [114,120,146] significantly reduces the aggregate available time, perhaps by one or more orders of magnitude. In spite of climate models, a variety of evidence indicates that local aqueous activity has occurred in Gale Crater well into the Amazonian, up until at least 2 Ga [271,297,298] and perhaps as recent as a few hundred Myr [299].

Studies of Garu crater and its vicinity suggest multiple crater lakes interconnected by hydrologic systems, including Gale crater, in late Hesperian times [106]. Modeling of the effects of obliquity cycling indicate recurring transient liquid wet conditions are possible on the Myr timescale [300]. Even if a new OoL did occur in the foreseeable future, however, the continued change in obliquity would revert to the conditions of today, which are considered inclement except at km scale depths [301,302].

### 4.2. Where an OoL Would Have Been on Mars

Crater-forming impacts continue to this day [303], at the rate of at least 700 per decade, some revealing shallow, extensive ice [304]. For contemporaneous Mars, however, there appears to be a major lack of opportunities for an OoL, and even daunting challenges for the survival of the most highly evolved extremophiles, because of the sub-freezing environment and the oxidative reactants in the atmosphere and soil [304]. Hydrothermal zones would be promising candidates, but would need to have a mechanism of recharge of liquid H_2_O, which seems difficult under the broad-scale current thermal conditions, even with a substantial subsurface hydrosphere [141].

Volcanism persisted into the Amazonian and may still occur, making the detection of geothermal anomalies, such as those mapped from orbit at Earth’s Yellowstone caldera [305], of particular interest. However, systematic observation campaigns by the Thermal Emission Imaging System (THEMIS) on the Mars Odyssey mission with its 100 m scale footprint, 2 K thermal sensitivity, and complete global coverage, have not revealed any locations on Mars where elevated surface temperatures might indicate the local availability of geothermal heat (V. E. Hamilton, personal communication, 2021).

After the discovery by the Viking missions of the unexpected paucity of organic compounds in the surface soil on Mars, it was realized that photochemically generated oxidants, including H_2_O_2_, OH·radical, atomic O_1_, peroxy radicals (HO_2_), superoxide ions (HO^2−^), and less reactive O_2_ itself, provide a significantly oxidative environment that can destroy [109,111] or degrade organics to unreactive carboxylate derivatives [306].

Contemporary Mars is presumably inhabitable at km-scale depths where temperatures can be high enough due to the planetary geothermal gradient to support a liquid hydrosphere [141]. However, habitability is not expected in the near surface where it is too cold, too dry, and too susceptible to damaging GCR radiation from space [301]. Future Mars actually holds some promise of an OoL because of upcoming favorable obliquity cycles, with the possibility of cold traps becoming sufficiently warmed to melt ice during daytime [300,307]. Could a macrobiont or nascent biosphere survive long-term when obliquity returns to unfavorably low values and the near-surface again becomes frozen and generally uninhabitable?

A most restrictive factor is the dearth of reducing power on Mars today. Hydrogen gas in the atmosphere is now only 15 ppm [308], which is too low for exergonic reactions with the abundant sulfates or atmospheric CO_2_, and the organics in soils are also measured in ppm, as noted above.

### 4.3. Likelihood for Origin of Life

With the present state of knowledge, it is difficult to assign which planet, Mars or Earth, originally provided the greater a priori likelihood for an OoL. Mars was perhaps simply too dry and too cold, for too much of the time. Or, it was too small and therefore too inactive (volcanically and geomagnetically). In contrast, perhaps the Earth’s surface was too wet and had too little sulfur, for life to arise in the first geological instant after the sterilizing bombardments waned. Perhaps Earth had too little boron available in its early history [309], whereas Mars did, which reinforces previous speculations for lithopanspermia, manifested as a Martian origin for life transplanted life to Earth [10,16,310,311,312,313,314].

At the most fundamental level, there is the “H_2_O Problem.” All scenarios for the origin of the form of life we know about have a requirement for the significant availability of H_2_O in the liquid phase. An excess of water, however, can result in too extreme a dilution of ingredients to support a successful PCE because of the much lower reaction rates needed to achieve transitions to avoid deleterious degradation rates of labile ingredients (e.g., hydrolysis).

A Chicxulub-class cometary impactor of diameter 30 km, with specific density of 1.0 and 10% soluble organics would provide only an average 4 µM concentration of organic molecules (at 100 g/mol) into a 3.5 km global equivalent layer (GEL) ocean on Earth. In comparison, the same bolide onto Mars where the total surface water inventory is, say, 0.5 km GEL [111], could produce a 100 µM concentration if those same organics were taken up by the water.

Or, perhaps neither planet qualifies as fully optimized for the rise of life, but something more intermediate between the two planets would have been even more favorable. If so, the very fact that it did arise gives hope, in the Bayesian sense, that the origin of life is not a formidable task, considering the panoply of settings that would be possible on any planet whose expanse is vast, endowed with essential elements and organics, and with the suitable environments for the formation of macrobionts of one class or another.

#### 4.3.1. Expected Value for the OoL

Expected value, and not just a probability, is the gauge for the likelihood of an origin of life on any given body. We currently cannot rule out any of the major hypotheses for the types of settings most suitable for life to have originated. Rather, it is possible that all are somewhat likely to have provided a pathway to life. Perhaps life began on one planet via one route, and on another planet by one of the other routes, depending on the relative prevalence of the various settings, and on happenstance.

Our agnostic approach, then, is to consider multiple plausible possibilities. If P_J_ is the probability of life beginning in a Jth type of setting, and N_J_ is the total number of settings of type J, then the expected value for an OoL in such a setting, E_J_[O], is simply.

E_j_[O] = N_J_ × P_J_ (1)

Even if the possibility of the chain of events leading to life is small yet non-negligible, but the number of settings in which it could occur is extremely large, then the expected value for an origin could be of order 1.0 or even higher.

Let us also assume, for sake of analysis, that there are four mutually independent *types* of proto-macrobionts where life could begin. Since they are disjoint, their probabilities and expected values are simply additive:

E[O] = E_PAT_[O] + E_GHS_[O] + E_OHV_[O] + E_X_[O] (2)

where PAT denotes a pond at ambient temperatures, GHS is for geothermal hot springs, OHV is oceanic hydrothermal vent, and X is “other” settings we have not yet considered or have been even conceived. Note that both impact crater lakes with hydrothermal consequences and magmatically heated groundwater could be subsets of GHS. Likewise, two or more types of smokers, or a vent field in general, could make up the OHV category. Thus, the number of suitable loci for an OoL could be even greater than the four indicated above.

The above considerations neglect the parameter of time. Proto-macrobionts come and go, e.g., because of the limited active lifetimes of ponds, springs, and hydrothermal vents. The planet-wide occurrences and lifetimes are a function of climate and magmatic changes, and hence vary. If we take p_J_(n,t) as the probability per unit time that the nth setting of type J will become a macrobiont that seeds a biosphere, then more explicitly,

P_J_ = p_J_(n,t) dt (3)

and

N_J_     L_J_

E_J_[O] =   ∑   ∫ p_J_ (n,t) dt (4)

N = 1  0

where N_J_ is the number of sites of type J, each with its own probability function, and L_J_ is the average lifetime for that type of setting before becoming a macrobiont that achieves colonization. One other aspect is that although L_J_ is typically small compared to geologic time, a given setting may be “re-used.”

Although this is the simplest possible model, it does emphasize that, for J-type settings, which are quite numerous (N_J_) and have sufficient typical lifetimes, L_J_, the probability can be quite small yet yield a reasonable expectation that one of more biosphere-seeding macrobionts can succeed, albeit rarely so on an individual basis. Including finite lifetimes in the formulation also emphasizes that, even when a given setting proceeds on a non-productive course, it will eventually be changed, sometimes for the better. For example, ponds can be covered over, springs can dry up, and vent chimneys clogged. However, they can also be rejuvenated, just as a test tube in the lab can be rinsed and re-used, which multiplies the opportunities for repeated “experiments” along the pathway to life and a biosphere.

#### 4.3.2. Lithopanspermia

If the expected value is so extremely low that the OoL on Earth is an outlier event for planets of our general type and located favorably in their planetary system, then any expectation for an additional, independent origin of life on Mars must be negligible (multiplication of two very small probabilities). However, given the possibility that life arose on Mars early, could it have also seeded Earth (or vice versa)?

When Mars-to-Earth lithopanspermia was first proposed, as a result of the confirmation that SNC meteorites had been successfully transported from Mars without melting from the shock acceleration to escape the gravity of Mars, it was also realized that there could be several impediments that would render the transfer of life a very low probability event [310,311]. These impediments include the energetics of launch at Mars, the statistics of capture by Earth, and the insults of space radiation from solar particle events and GCR [312,313]. The rocks launched during the spallation process must generally be highly competent, as evidenced by the population of known Martian meteorites, which implies difficulty if not impossibility in launching the weaker sediments which would typically contain the much higher and more diverse bioloads. Once the spall phenomenon explanation was established, for the transfer mainly of kinetic rather than thermal and disruptive energy by an impacting bolide into rock in a spall zone [315], it became clear that seeding the Solar System was possible. However, detailed calculations of the statistics of interplanetary transfer [316] showed that such events required significant transfer times, such that the disruption of biological organization and processes by ionizing space radiation (penetrating GCR) could be too severe in all but the less likely cases of multi-meter scale meteorites [313]. Transfers from Earth to Mars are significantly less likely [316] because of Earth’s much higher gravity field and thicker atmosphere, and the smaller target provided by Mars (smaller diameter, larger orbit).

From a probabilistic standpoint, Mars-to-Earth panspermia seems extraordinarily unlikely because it presumably is the multiplicative product of potentially two very small numbers, i.e., the probability of an OoL on Mars and the probability of successful transfer and colonization of Earth.

However, even if it is an extremely rare outcome, once life started on Mars, the large number of transfers that intrinsically occur, especially in the earlier high bombardment rate history of the solar system, could balance against the low probability of successful transfer, to combine for a likelihood for seeding Earth that is not necessarily out of the question [313]. Given that the opportunities for an OoL on Mars were highest in the time interval just as life appeared on Earth could be a coincidence, or it could be because Mars had the greater a priori advantage for the OoL. Detecting past life on Mars would be extraordinarily important for many reasons, but including the capability for comparative genomics to assess whether there were two OoL’s which were truly independent, and if not, to determine the locus in the evolutionary tree of life where the branching took place and to constrain when the migration occurred based on the genomic clock.

### 4.4. Future Research

Mars orbiter missions will continue to make major contributions, but rovers on the surface are particularly well-suited for discoveries that can be confirmed only with the analyses that are possible in situ, as well as mineralogical confluences and aqueous settings that are subscale or otherwise not detectable from orbit.

Much remains to be learned of Mars from the bountiful number of operational and new missions (NASA’s Perseverance and CNSA’s Zhurong rovers) to the planet. These missions can take opportunities to explore with high relevance to the field of OoL, depending on how operations are implemented, especially with respect to the selection of samples to be returned for extremely detailed analyses in laboratories on Earth.

Mars sample return missions are often justified because of the lack of geologic context for the hundreds of Martian meteorites that have now been found on Earth. These meteorites are generally composed of igneous minerals, with only occasional minor or trace quantities of alteration products. Because the ejection process is energetic, converting plagioclase to maskelynite, with indications of peak shock pressures of 20 to 80 GPa [317], it is unlikely that sedimentary rocks such as mudstones and sandstones can be successfully ejected without becoming disaggregated. Sediments can not only establish the record of aqueous processes, and hence the past habitability of Mars [8,318,319], but are also the medium in which the secondary minerals and organics for PCE and the OoL itself reside. Hence, it is advocated that promising sediments be given high priority in the upcoming selection of samples by the M2020 Perseverance mission for future return to Earth because they are much more likely not only to preserve life or biosignatures, but also the history of aqueous processes and PCE. This would be especially important if a sediment could be associated with one of the scenarios proposed for the OoL, such as hydrothermal or cyclic wet–dry settings. Laboratory investigations of such samples should include study of the composition of aqueous extracts for various pH/Eh conditions.

## 5. Summary

We have endeavored to demonstrate the numerous suitable circumstances at Mars for an origin of life. A synthesis combining the discoveries from the exploration of Mars with terrestrial analogs, relevant laboratory experiments, and theoretical models point towards an OoL on Mars as being likely to the same degree, and even more so in many respects, than the origin of life on Earth itself. Several of the discoveries of the MSL Curiosity rover mission in Gale crater, such as enrichments of key elements proposed for the OoL (Cu, B, etc.), direct evidence of cyclic wet–dry cycling (e.g., mudcrack patterns, episodic wetting), and the preservation of organics at the surface, are directly favorable to the likelihood of an origin of life.

Mars not only has all the building-block elements (CHNOPS) for biochemical molecules but also other key elements critical for metabolic functions, in many cases enriched over their abundances in rocks and soils on Earth. Several extremely important elements of biology, including sulfur, iron, and magnesium, are especially highly abundant and mobile on Mars, more so than on terrestrial continents. Furthermore, several transition trace elements which serve as co-factors in important metalloenzymes, such as Mn, Ni, and Zn, are also unusually abundant. Ubiquitous amorphous components in Martian sediments are additional evidence of element mobility. Whether each of these elements are available in their most suitable form (solubility and redox state) depends on the pH and Eh of the contemporaneous environment, but these can be modulated by the intensity and duration of local mineral alteration or magmatic activity and attendant release of volatiles.

Settings suitable for the various OoL hypotheses are abundant. The extreme population of crater basins fulfills pond scenarios for macrobiont formation. The proximity to the Kuiper and outer asteroid belts assures equal or greater contribution than for Earth of the carbonaceous matter and pre-formed biotic precursors these contain, as well as accessible sources of phosphorus and nickel, while the shallower regolith, less water, and less active surface processes allow for greater concentrations of these components.

Although suboceanic hydrothermal vents were undoubtedly much less common on Mars because of less water, little or no plate tectonics, less magmatic activity, and uncertainties about the extent of an early ocean, if present at all, there is nonetheless widespread evidence of hydrothermal environments in the past. Furthermore, the global distribution of large craters provides the basis for meaningful durations of buried hydrothermal regimes created from impacts by large bolides, even if the initial regolith inventory of H_2_O was as ice.

Late Noachian and early Hesperian Mars were sufficiently endowed with periods of liquid water that life could have begun. This time period overlaps Earth’s Hadean and early Archean eon’s, during which life appeared. If wet–dry and/or freeze–thaw cycling are indeed critical environments to enable the prebiotic chemical evolution needed to achieve the reproduction of protocells and beyond, then Mars would have a significant advantage in its area of land compared to the surface area of rare volcanic islands in a global ocean envisioned at the time of early Earth. For freeze–thaw cycling, the arid environments which may have been much more prevalent on Mars than Earth generally experience significantly larger diurnal temperature swings, promoting the freezing of shallow streams and foreshore areas at night, but with melt-out in daytime.

The period for an OoL on Mars from late Hesperian to current epochs would be less favorable on the basis of the rarity of liquid H_2_O. However, it was in earlier times that life was already apparent on Earth. Given that life could have arisen on either planet, and with the interchange of ejected material from hypervelocity impact, it is possible that one planet seeded life on the other. For a variety of reasons, the expected probability of Mars-to-Earth lithopanspermia is greater than for the opposite direction.

Although the outlook for a future OoL is bleak, Mars is serving as a window into plausible conditions on early Earth, a time period in our geologic history which has been erased by subsequent processes. It also is providing support for hypotheses which view suitable exoplanets as candidates for their own origin of life. Further exploration by the 2020 and subsequent rovers will undoubtedly expand the list of relevant conditions and constituents that have occurred on Mars.

Given that sediments are generally too weak to be ejected from Mars by natural impact processes, sample-return missions could greatly enhance the value of laboratory analyses if sediments significantly populate the samples taken for potential return. Based on the range of settings hypothesized for the OoL, sediments collected from areas where spatially heterogeneous or time-variable conditions are in evidence may be especially beneficial for gaining insights into prebiotic chemical evolution and the steps leading to life.

## Abbreviations

AmC	Amorphous Component (ChemCam XRD)
APXS	Alpha Particle X-ray Spectrometer (XRF on MER, MSL rovers)
a_w_	Chemical activity of H_2_O
CCAM	ChemCam (MSL/LIBS on MSL rover)
CheMin	Chemical and Mineralogy instrument (XRD on MSL rover)
CRISM	Compact Recon Imaging Spectrometer for Mars (MRO orbiter)
OMEGA	Vis/IR Mineralogical Mapping (ESA/Mars Express orbiter)
EGA	Evolved Gas Analyzer (part of SAM on MSL rover)
GHS	Geothermal Hot Spring
GCMSGas	Chromatograph and Mass Spectrometer (Viking, MSL/SAM)
GCRGalactic	Cosmic Radiation
LAWKI	Life As We Know It (biochemically)
LIBS	Laser-Induced Breakdown Spectroscopy
MERMars	Exploration Rover (Spirit and Opportunity rovers)
MORB	Mid-Ocean Ridge Basalt
MS	Mass Spectrometer
MSL	Mars Science Laboratory (Curiosity rover)
M2020	Mars 2020 Mission (Perseverance rover)
NWA	Northwest Africa (meteorite identifier)
OoL	Origin of Life
OHV	Oceanic Hydrothermal Vent
PAT	Pond in Ambient Temperature
PIXL	Planetary Instrument for X-ray Lithochemistry (M2020 rover)
PCE	Prebiotic chemical evolution
RNAzyme	RNA enzyme (ribozyme)
SAM	Sample Analysis at Mars (GCMS, EGA on MSL rover)
THEMIS	Thermal Emission Imaging System (Odyssey orbiter)
WCL	Wet Chemistry Laboratory (instrument on Phoenix lander)
XRD	X-ray Diffraction
XRF	X-ray Fluorescence
